# Ecosystem Functions across Trophic Levels Are Linked to Functional and Phylogenetic Diversity

**DOI:** 10.1371/journal.pone.0117595

**Published:** 2015-02-18

**Authors:** Patrick L. Thompson, T. Jonathan Davies, Andrew Gonzalez

**Affiliations:** Department of Biology, McGill University, Montreal, Quebec, Canada; Chinese Academy of Sciences, CHINA

## Abstract

In experimental systems, it has been shown that biodiversity indices based on traits or phylogeny can outperform species richness as predictors of plant ecosystem function. However, it is unclear whether this pattern extends to the function of food webs in natural ecosystems. Here we tested whether zooplankton functional and phylogenetic diversity explains the functioning of 23 natural pond communities. We used two measures of ecosystem function: (1) zooplankton community biomass and (2) phytoplankton abundance (*Chl a*). We tested for diversity-ecosystem function relationships within and across trophic levels. We found a strong correlation between zooplankton diversity and ecosystem function, whereas local environmental conditions were less important. Further, the positive diversity-ecosystem function relationships were more pronounced for measures of functional and phylogenetic diversity than for species richness. Zooplankton and phytoplankton biomass were best predicted by different indices, suggesting that the two functions are dependent upon different aspects of diversity. Zooplankton community biomass was best predicted by zooplankton trait-based functional richness, while phytoplankton abundance was best predicted by zooplankton phylogenetic diversity. Our results suggest that the positive relationship between diversity and ecosystem function can extend across trophic levels in natural environments, and that greater insight into variation in ecosystem function can be gained by combining functional and phylogenetic diversity measures.

## Introduction

After two decades of biodiversity-ecosystem function research, there is now consensus that the functioning of a biological community is mediated by the diversity of its component species [1,2]. Most experiments reveal that ecosystem function has a positive but saturating relationship with species richness [2]. However, in experimental data, species richness typically accounts for between 30 to 73 percent of the variance of a given ecosystem function [3]. This wide range has prompted ecologists to look for measures of diversity that more reliably explain variation in ecosystem function, including estimates of functional and phylogenetic diversity [4–6].

Measures of functional diversity are typically based on a subset of traits of the component species that are known to be important for ecosystem functions [7]. In general, such measures require careful *a priori* consideration of which traits to include, and whether or not traits should receive different weights. Despite these complications, functional diversity measures often better explain variation in ecosystem function than species richness and other taxonomic diversity measures [8,9].

Another approach has been to relate the phylogenetic diversity—a measure of the evolutionary relatedness of species in a community—to ecosystem function [10]. This relies on the hypothesis that closely related species are more functionally similar than distantly related species, and therefore a more phylogenetically diverse community will have greater functional complementarity. While this will not be the case if functional traits show convergence in the phylogeny, two recent studies found phylogenetic diversity to be a better predictor of ecosystem function than species richness, and an equal or better predictor than indices incorporating functional traits [9,11]. An advantage of using phylogenetic diversity is that it can capture functional differences due to unmeasured or immeasurable traits, and is more readily applicable to groups such as microbes, where traits are less often measured [10].

To date, studies relating functional and phylogenetic diversity to ecosystem function have been largely limited to experimental settings [9,11,12], but see [13]. Therefore, it is unclear whether these diversity measures will still be strong predictors of ecosystem function in natural communities, where environmental conditions are not controlled as they are in experiments.

Furthermore, the use of functional and phylogenetic diversity measures for predicting ecosystem function has been focused on productivity in plant communities [10], but see [13–15]. However, we expect that these measures should also improve our understanding of the functioning of higher trophic levels [16,17]. Experimental evidence suggests that more species-rich herbivore assemblages are 1) able to exert stronger top-down control on plant biomass, and 2) able to produce greater herbivore biomass [3,18]. This effect may be because communities with more herbivore species are likely to graze on a variety of plant types. If the ability, or preference, to graze on certain plant types is linked to traits, then measures of the functional and phylogenetic diversity of herbivores should explain more variation in the strength of top-down control compared to species richness. Of course, plant biomass may also be influenced by bottom up effects, such as nutrient availability, which may obscure the top down effect of grazer diversity [19]. Regardless of these complications, testing whether functional and phylogenetic diversity measures are predictive of the functioning of herbivores in complex natural communities is an obvious next step.

**Fig 1:**
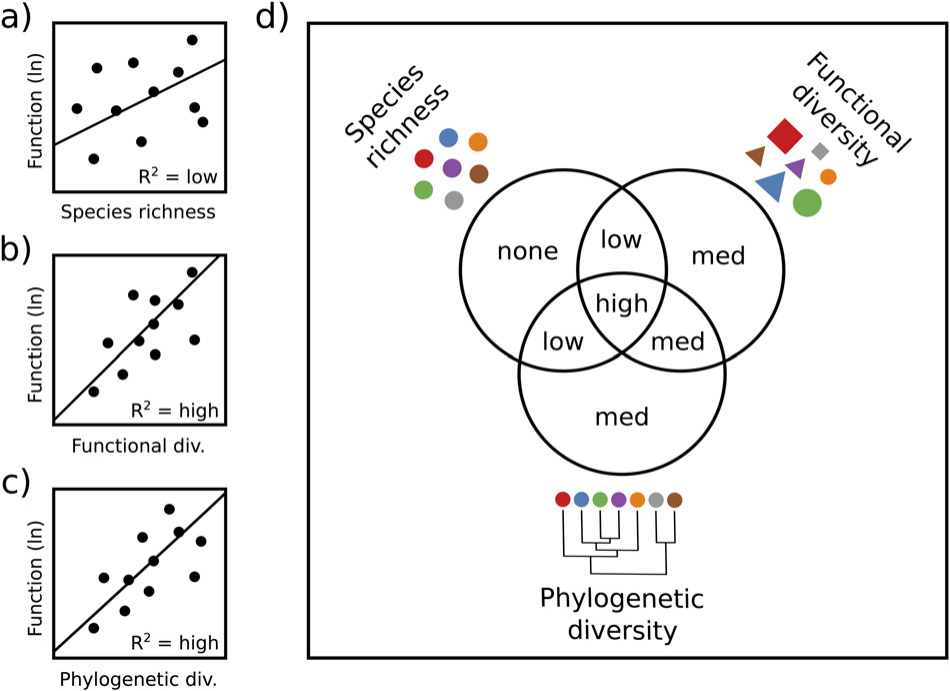
Hypothesized relationships between ecosystem function and species richness (a), functional diversity (b), and phylogenetic diversity (c). We predict a stronger relationship with ecosystem function, and thus a higher R^2^, for functional diversity (b) and phylogenetic diversity (c) than for species richness (a) because the former two measures incorporate information about the traits, or the evolutionary similarity of the different species in the community. Panel (d) depicts the results of variation partitioning, indicating our hypothesis that functional and phylogenetic diversity will explain all of the variation explained by species richness, as well as additional variation, both overlapping and unique.

To address whether functional and phylogenetic diversity are useful predictors of ecosystem function for food webs in natural communities, we examined the explained variance in the functioning of natural pond communities across two trophic levels. We used two measures of ecosystem function, (1) zooplankton community biomass and (2) phytoplankton abundance (*Chl a*), to test for diversity-ecosystem function relationships within and across trophic levels. We predicted that more diverse zooplankton communities would have higher biomass and would exert stronger top-down control, suppressing phytoplankton abundance. Furthermore, because diet complementarity is likely related to the diversity of functional traits or phylogenetic relatedness, we predicted that both functional and phylogenetic diversity would overlap in the variation in ecosystem function explained, but each might explain additional variation beyond that captured by species richness alone (Fig. 1). Detecting these relationships is complicated by the fact that environmental conditions are not constant across ponds, and this can affect both diversity and ecosystem function. Therefore, we used structural equation modelling to explore the direct and indirect effect of environmental gradients on the diversity-ecosystem function relationships [20].

## Materials and Methods

### Pond Zooplankton Survey

We conducted a survey of 23 ponds in the Gault Nature Reserve (GNR), Quebec, Canada (45° 32’ 10” N, 73° 09’ 10”, W) on May 23–27, 2011. GNR is a UNESCO Biosphere Reserve, and one of the few remaining examples of primeval forest in the region. Permission for conducting this survey was given by the director of the GNR. This study did not involve endangered or protected species, or any vertebrate species. The ponds were a mix of ephemeral and permanent, and were scattered around the 1000-hectare reserve (S1 Fig.). They ranged in size from 0.0006 to 0.136 hectares and from 0.11 to 0.60 meters deep, and spanned an elevation gradient of 204 to 415 meters above sea level. All ponds were surrounded by similar forest habitat, and had low observed macrophyte abundance. The benthic habitat of these ponds was characterized by very loose sediment, so that the sediment water interface was not well defined.

Our survey determined zooplankton community composition, phytoplankton abundance (*Chl a*), and relevant abiotic variables in each pond. All samples for water chemistry, zooplankton, and phytoplankton were collected from the centre of the pond using a 4L horizontal VanDorn water sampling bottle (Wildco, USA). The process of collecting the samples from such shallow ponds inevitably caused mixing of the water column so that the samples contained water, zooplankton, and phytoplankton from all depths.

Total phosphorous (TP), dissolved organic carbon (DOC), and dissolved inorganic carbon (DIC) concentrations were analysed by the GRIL Aquatic Analytical Laboratory (UQAM, Montreal). Dissolved oxygen (DO), and pH were measured using a handheld probe (YSI, USA). Elevation and pond surface area were measured using a handheld GPS (Garmin, USA). Depth was measured in the centre of each pond using a meter stick. Canopy cover was estimated visually. Pond permanence was estimated as the number of days after sampling that the ponds retained water, to a maximum of six months. This measure of permanence was based on temperature sensors, placed at the bottom of each pond, taking measurements every 30 minutes. Temperature time series were inspected visually, and the pond was assumed dry when the amplitude of the daily temperature fluctuations increased by at least twice. These measurements were corroborated with monthly visits to the ponds. We measured conductivity but we were unable to include it in our analysis because our probe was malfunctioning and did not provide readings for all of our ponds.

### Zooplankton community survey

Zooplankton samples were collected by passing 8–16 L of water through a 75 μm Nitex sieve. Higher volumes were sampled when zooplankton appeared to be in low abundance. Zooplankton were anesthetized using carbonated water and preserved in 95% ethanol.

Crustacean zooplankton were enumerated and identified using a dissecting microscope at 60x magnification (Leica, Germany). Entire samples were counted for all species (mean of 1116 and median of 690 individuals per sample), and identifications were performed using a compound microscope (Leica, Germany) when necessary. All organisms were identified to the highest possible taxonomic resolution, generally species, according to Haney [21]. For our analyses, we used genus level distinctions, to maintain consistency across all taxa, with the exception of harpacticoid copepods, which were left as a group because their identification is challenging. None of our genera contained more than one identified species and so this taxonomic resolution appears to be an accurate representation of the diversity within these ponds. It remains possible that we may have classified multiple species together when we were unable to identify to species level (e.g., the harpacticoid copepods), but based on the morphological similarity of individuals, we expect this underestimation of diversity to have been minimal. We were unable to identify copepod nauplii to species, but we included them in our estimates of community biomass. Biomass was estimated using average measured body lengths and length-weight regressions [22], multiplied by the number of individuals and divided by the volume of water sampled.

### Ecosystem functions

Zooplankton community biomass was calculated as the summed biomass of all taxa per litre of sampled pond water. The top down effect of zooplankton on phytoplankton abundance was estimated as the relationship between zooplankton diversity and chlorophyll *a*; thus the highest zooplankton function occurs when chlorophyll *a* is lowest. Chlorophyll *a* concentrations were estimated by filtering at least 250 mL of water, through a GF/F filter paper. Chlorophyll concentrations (μg L^-1^) were analysed spectrophotometrically after cold extraction in ethanol using the acidified method [23]. It was not possible to directly sample for periphyton because of the loose sediment water interface, but observations suggest that it was not abundant. Therefore, it is unlikely that disturbance of periphyton during sampling would have contaminated our phytoplankton samples.

### Diversity Indices

Three taxonomic diversity measures, species richness (SR), Shannon diversity (Shan), and Simpson diversity (Simp), were calculated using the *vegan* package [24] in R version 3.0.2 [25]. Rarefaction curves were produced using the *vegan* package to evaluate whether our estimate of SR was affected by the number of zooplankton in our samples, and thus variation in the abundance of zooplankton across the ponds. There are a variety of indices for calculating functional and phylogenetic diversity, each emphasizing a different aspect of the diversity of a community [26,27]. Therefore, using multiple complementary indices can provide insight into how ecosystem function is dependent upon different aspects of community diversity.

We chose to use a set of three independent functional diversity measures that each capture one of the three primary components of functional diversity: richness (FRic), evenness (FEve), and divergence (FDiv)[28]. These were calculated using four zooplankton traits (body length, feeding type, habitat preference, and trophic group) from Barnett et al. [29] and unpublished updates from Beisner et al. (in prep), using the *FD* package [30]. We selected these traits because they affect feeding and biomass either directly (body length, feeding type, trophic group) or indirectly (habitat preference—reflective of which parts of the ponds zooplankton can graze phytoplankton and produce biomass). Habitat preference consisted of three categories: littoral, intermediate, and pelagic. Trophic group consisted of four categories: herbivore, omniherbivore, omnivore, omnicarnivore. The trait for feeding type consisted of raptorial feeding, C-filter feeding (scraping and filtering), D-filter feeding (stationary feeding with filtering apparatus on 3^rd^ and 4^th^ legs), and S-filter feeding (stationary feeding with filtering apparatus on legs 1–5) which we expect to determine the type of food particles caught [29]. Because we considered multiple filter feeding types we added a fifth trait, raptorial vs. filter feeding, to differentiate filter feeding from raptorial feeding. When calculating our measures of functional diversity, the new feeding type trait and the original feeding type trait both received half the weighting that we gave to each of the other traits. This allowed us to maintain equal weighting of the four initial traits. Including this additional trait to differentiate filter and raptorial feeding resulted in more logical functional associations between species (S2a Fig.) but did not significantly change our results (results excluding additional trait not shown). Only the trait for body size was numeric so the traits were not standardized. Traits were matched based on our highest level of taxonomic resolution (generally species).

FEve and FDiv were calculated as both presence-absence (_pa_) and abundance weighted (_ab_) measures. We also created a functional dendrogram based on Ward’s clustering method of the five traits (S2a Fig.)[7]. This dendrogram was used as a visual representation of the groupings of functional diversity but was not analysed directly as a measure of functional diversity [7]. We also compared the predictive efficacy of single traits with our multi-trait indices. For this, we calculated the functional richness of each individual trait for comparison with the functional diversity measures based on multiple traits. Functional divergence and evenness cannot be calculated for single traits.

We chose to use two phylogenetic diversity measures: Faith’s Phylogenetic Diversity (PD)[31], and standard effect size mean pairwise distance (sesMPD)[32]. PD provides a simple measure of the phylogenetic relatedness of a community based on the summed branch lengths of its phylogenetic tree. We expected this measure to capture functional complementarity well if more distantly related species are more functionally unique. However, PD is highly dependent upon species richness, so we also chose to use sesMPD, which provides a measure of phylogenetic diversity that is independent of species richness. sesMPD is equal to-1 times the net related index (NRI) [32] and was used instead of NRI because it increases with community phylogenetic diversity. PD and sesMPD were calculated using the *Picante* package [33], using the phylogenetic tree published in Helmus et al. [34] (S2b Fig.). Taxa were matched to the tree based on our finest level of taxonomic resolution (generally species). Harpacticoid copepods were not included on this tree and so were added, halfway between the calanoid and cyclopoid copepods, as was done by Helmus et al. [34] when sequence data was not available, and according to the taxonomic tree provided by Huys and Boxshall [35]. sesMPD was calculated as both presence absence (_pa_) and abundance weighted (_ab_).

### Statistical Analysis

We used linear regression to test for relationships between ecosystem function and our explanatory variables. Type II linear regression, with the ranged major axis method to account for error in both the independent and dependent variables using the *lmodel2* package [36], was used to determine the slope of relationships, unless a polynomial term was included, in which case a Type I regression was used. Response variables were ln transformed to improve normality. The diversity measure of each type (taxonomic, functional, and phylogenetic) that best explained ecosystem function was selected based on the Akaike Information Criterion (AIC). Overlap in variance explained by the best performing diversity measures of each type was evaluated using variation partitioning in *vegan* [24]. The correlation between traits and phylogeny was calculated using collectively using a Mantel test [37] in *vegan*. Phylogenetic signal for each individual trait was tested using Blomberg’s K statistic of phylogenetic signal in *Picante* [33] for continuous traits (body size), using the phylogenetic D statistic [38] in the *caper* package [39] for binary traits (raptorial vs. filter feeding), and using Pagel’s λ [40] in the *GEIGER* package [41] for multistate traits (habitat, feeding type, and trophic group).

Last, we explored effects of environment on community diversity and ecosystem function together using structural equation modelling (SEM) [42]. This allowed us to determine if our diversity-ecosystem function relationships were a product of both co-varying with environment, or if there was an independent and direct effect of diversity on the ecosystem functions as hypothesized. We started with a model involving all plausible pathways between environment, our best diversity measures (identified through linear regression), and the two ecosystem functions (S3 Fig.). We then compared this model to simplified models where pathways had been removed and used model selection based on AIC to determine the best fit model of the two ecosystem functions. If the pathway between diversity and the ecosystem function remains significant when environment is allowed to affect both diversity and the ecosystem function, we can conclude that there is an independent effect of diversity on the ecosystem function. We compared models including three combinations of our environmental variables: 1) total phosphorous (only for predicting chlorophyll *a*); 2) subsets of environmental variables determined as important predictors of each function based on multiple regression and model selection based on comparing all variable combinations using the *leaps* package [43]; 3) the first two axes from a PCA of all standardized environmental variables (S4 Fig.) calculated using the *vegan* package [24]. The first two axes of the PCA contained 57.1% of the variation in the environmental variables. All analyses were performed in *R*, version 3.0.2 [25].

## Results

### Zooplankton community characteristics

Average pond species richness was 4.42, ranging from 2 to 7, with a regional richness of 10. Across all ponds, zooplankton community biomass was 249.57 μg L^-1^ on average, ranging from 0.25 to 938.04 μg L^-1^. Species richness was positively, albeit weakly, related to the number of zooplankton present in our samples (R^2^ = 0.29; *p* = 0.006). However, the rarefaction curves saturated in the majority of samples (87%), suggesting that our estimates of richness were not greatly biased by differences in the abundance of zooplankton amongst the ponds (S5 Fig.).

*Daphnia pulex* comprised 51.3% of the zooplankton biomass over all ponds and was present in 14 of the 23 ponds. The next most abundant genus, *Microcyclops rubellus*, comprised 15.8% of the zooplankton biomass in all ponds and was present in 18 of the 23 ponds. *Acanthocyclops vernalis* comprised 12.1% of the zooplankton biomass in all ponds and was present in 15 of the 23 ponds. This species is carnivorous as an adult but was retained in our analysis because it consumes phytoplankton in its juvenile stages [44]. There were seven rarer taxa, *Alonella sp*., *Ceriodaphnia dubia*, *Chydorus sphaericus*, Harpacticoida, *Sida crystallina*, *Simocephalus sp*., and *Tropocyclops prasinus mexicanus*, that each made up less than 5% of the average biomass, and all but Harpacticoida were negatively correlated with *D*. *pulex* abundance. Phytoplankton abundance was 6.56 μg chl *a* L^-1^ on average, ranging from undetectable to 33 μg L^-1^. Zooplankton species richness was not related to either chlorophyll *a* (R^2^ = 0.07; *p* = 0.211) or total phosphorous (R^2^ = 0.10; *p* = 0.134).

The combined zooplankton traits were closely correlated with their phylogeny (S2 Fig.; Mantel test, *r* = 0.852, *p* = 0.001, 999 permutations). Feeding type and our raptorial vs. filter feeding traits showed significant phylogenetic signal (feeding type—χ^2^ (1) = 7.54, *p* = 0.006;; raptorial vs. filter feeding—D = -3.73, *p* < 0.001), indicating phylogenetic conservatism for these traits. The body length, habitat preference, and trophic group traits did not show significant phylogenetic signal (body length—K = 0.84, *p* = 0.172; habitat preference—χ^2^ (1) = 0.004, *p* = 0.950, trophic group—χ^2^ (1) = 2.52, *p* = 0.112), indicating little or no phylogenetic conservatism for these traits.

### Zooplankton community biomass

**Fig 2:**
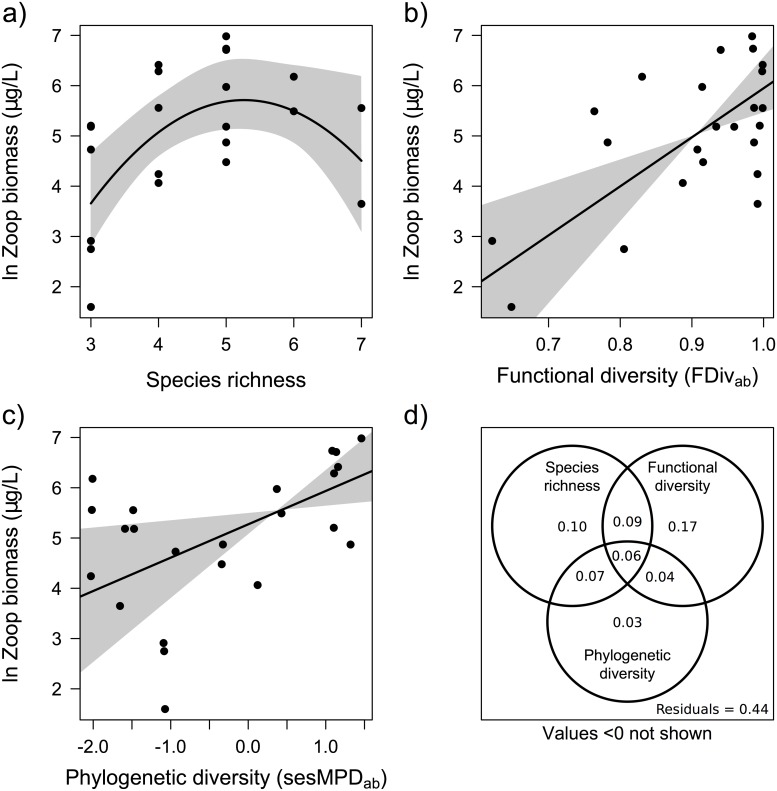
Zooplankton community biomass in the 23 ponds as predicted by the best diversity indices in each category: taxonomic diversity—species richness (a), functional diversity—abundance weighted functional divergence (b), phylogenetic diversity—abundance weighted standard effect size mean pairwise distance (c), and the variation partitioning for these three models with their adjusted R^2^ (d). Significant model trends are shown as black lines. The grey bands indicate the 95% confidence intervals for the predicted values (a) and the slope of the regression lines (b,c).

Five out of the 11 diversity measures tested explained a significant proportion of variance in zooplankton community biomass, and in all cases, there was a positive influence of diversity on biomass (Table 1). These significant models included taxonomic, functional, and phylogenetic measures. Abundance weighted functional divergence (FDiv_ab_), explained the most variance of any diversity measure (Fig. 2b; R^2^ = 0.39; *p* = 0.001). Species richness (SR), explained the second most variance, although it exhibited a unimodal relationship with zooplankton community biomass, where the highest biomass was found in ponds with intermediate species richness (Fig. 2a; R^2^ = 0.38; *p* = 0.008). This unimodal relationship between species richness and community zooplankton biomass outperformed a model that assumed a linear relationship (R^2^ = 0.14, *p* = 0.077). Abundance weighted standard effect size mean pairwise distance (sesMPD_ab_) explained the third highest proportion of variance of the single diversity measure models (Fig. 2c; R^2^ = 0.23; *p* = 0.019).

**Table 1:** Results of the linear models for predicting zooplankton community biomass (ln), ranked in increasing order of AIC.

	variable	type	d.f.	AIC	ΔAIC	R^2^	R^2^ Adj	slope	P
1	FDiv_ab_ + Env*	other	6	70.6	0	0.585	0.493	-	0.002
2	FDiv_ab_	functional	3	73.5	2.9	0.388	0.359	9.875	0.001
3	Trophic group	1 function	3	74.9	4.3	0.351	0.321	1.258	0.003
4	Raptorial vs. filter	1 function	3	75.5	4.9	0.334	0.303	1.688	0.004
5	Env.*	other	5	75.6	5.0	0.446	0.359	-	0.009
6	SR^2^	taxonomic	4	75.8	5.2	0.383	0.321	4.2190*x-0.4007*x^2	0.008
7	Body size	1 function	3	75.9	5.3	0.324	0.291	3.974	0.005
8	sesMPD_ab_	phylogenetic	3	78.7	8.1	0.234	0.198	0.677	0.019
9	PD	phylogenetic	3	79.0	8.4	0.212	0.186	0.002	0.023
10	FRic	functional	3	79.9	9.3	0.192	0.153	6.076	0.037
11	FDiv_pa_	functional	3	80.7	10.1	0.165	0.125	11.488	0.055
12	Feeding type	1 function	3	80.9	10.3	0.159	0.119	0.745	0.060
13	FEve_pa_	functional	3	81.3	10.7	0.142	0.101	7.150	0.076
14	SR	taxonomic	3	81.3	10.7	0.142	0.010	0.613	0.077
15	Env. PCA**	other	4	81.8	11.2	0.197	0.116	-	0.116
16	sesMPD_pa_	phylogenetic	3	82.8	12.2	0.084	0.040	0.839	0.180
17	Chl *a*	other	3	83.2	12.6	0.070	0.026	-0.673	0.221
18	Simpson	taxonomic	3	84.0	13.0	0.012	-0.035	-1.401	0.624
19	Shannon	taxonomic	3	84.7	14.1	0.006	-0.041	-0.660	0.723
20	FEve_ab_	functional	3	84.8	14.2	0.002	-0.045	0.337	0.833
21	Habitat type	1 function	3	84.8	14.21	0.002	-0.046	0.170	0.847

The highest ranked model of each diversity type is bolded. *P* values that are less than 0.05 are bolded.

* Environmental variable model includes elevation, DIC, and ln TP

** Environmental PCA includes first 2 axes of PCA on all standardized environmental variables.

Based on variation partitioning, SR, FDiv_ab_, and sesMPD_ab_ together explained 66% of the variation in zooplankton community biomass, and overlapped in explaining 6% of the variation (Fig. 2d). SR and FDiv_ab_ overlapped to explain 9% of the variation. SR and sesMPD_ab_ overlapped to explain 7% of the variation. FDiv_ab_ and sesMPD_ab_ overlapped to explain 4% of the variation. SR, FDiv_ab_, and sesMPD_ab_ uniquely explained 10%, 17%, and 3% of the variation respectively.

Three of the five traits (trophic group, raptorial vs. filter feeding, and body length) individually explained a significant amount of variance in zooplankton biomass but none outperformed the best functional diversity measure (FDiv_ab_) although ΔAIC was small (Table 1). These three single traits outperformed the best phylogenetic diversity measure (sesMPD_ab_).

The best performing model of environmental variables for predicting zooplankton community biomass consisted of elevation, DIC, and ln TP, and explained a significant amount of variation (R^2^ = 0.45; *p* = 0.009). However, when FDiv_ab_ was included in the model, none of these environmental variables remained as significant predictors, although the model outperformed that of FDiv_ab_ alone (Table 1). Chlorophyll *a* did not explain a significant amount of variance in zooplankton community biomass (R^2^ = 0.07; *p* = 0.221).

The pathway between FDiv_ab_ and zooplankton community biomass, was always significant, regardless of how we specified the effect of environment in our SEM (S6 Fig.). The most parsimonious model, based on AIC, only included the direct pathway from FDiv_ab_ (S1 Table). This suggests that our linear models adequately capture the relationship between diversity and zooplankton biomass.

### Phytoplankton abundance

**Fig 3:**
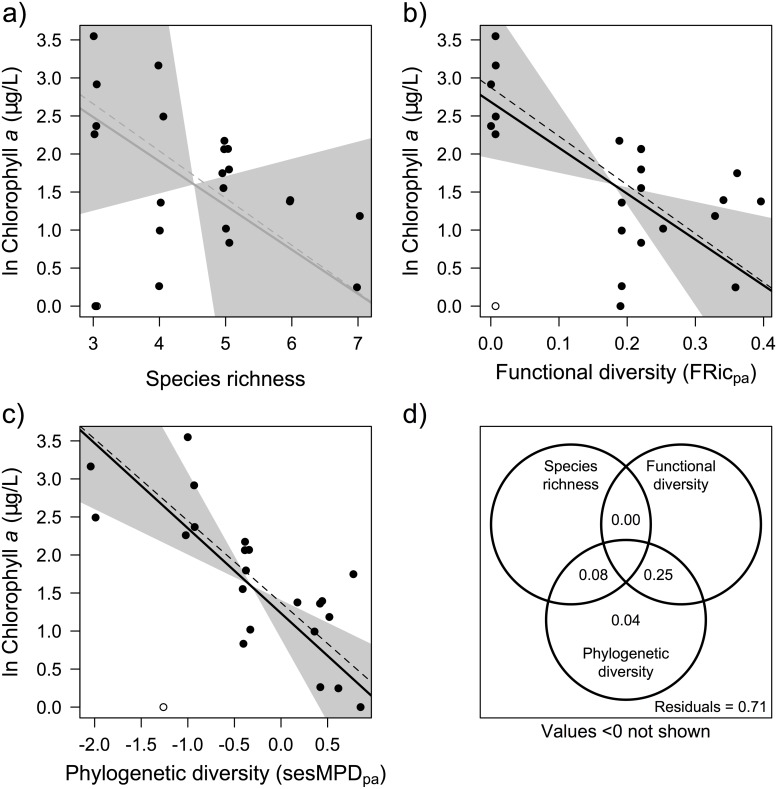
Chlorophyll *a* in the 23 ponds as predicted by the best diversity indices in each category: taxonomic diversity—species richness (a), functional diversity—functional richness (b), phylogenetic diversity—presence absence standard effect size mean pairwise distance (c), and the variation partitioning for these three models with their adjusted R^2^ (d). Significant model trends are shown as black lines. Insignificant model trends are shown as grey lines. The empty circles indicate the pond that is an outlier to the predicted trend. The dashed lines indicate the model trend when this outlier is removed. The grey bands indicate 95% confidence intervals for the slope of the regression lines.

Three out of the 11 zooplankton diversity measures explained a significant proportion of variance in chlorophyll *a*, and in all but one case, there was a negative influence of diversity on chlorophyll *a* (Table 2). These significant models included functional and phylogenetic, but not taxonomic diversity measures. The best single diversity measure for predicting chlorophyll *a* was the phylogenetic diversity measure sesMPD_pa_ (Fig. 3c; R^2^ = 0.38; *p* = 0.002). There was one outlier in this relationship, which was found to have significant influence (Cook’s distance > 0.5) on the analysis (Fig. 3c—unfilled point). Chlorophyll *a* was not detectable in this pond, although the predicted concentration should have been relatively high based on the measured zooplankton phylogenetic diversity. We cannot be sure if this chlorophyll *a* concentration is a measurement error, so we compared model fit with and without including it. Removing this outlier from our analysis did not have a large effect on the slope of the relationship but greatly improved the model fit (Fig. 3c, dashed line; R^2^ = 0.59, *p* <0.001).

**Table 2:** Results of the linear models for predicting chlorophyll *a* (ln), ranked in increasing order of AIC.

	variable	type	d.f.	AIC	ΔAIC	R^2^	R^2^ Adj.	slope	p
1	sesMPD_pa_	phylogenetic	3	58.4	0	0.376	0.345	-1.164	0.002
2	sesMPDpa + Env*	other	9	59.8	1.4	0.663	0.382	-	0.080
3	Raptorial vs. filter	1 function	3	60.9	2.5	0.301	0.267	-1.138	0.007
4	FEve_ab_	functional	3	62.3	3.9	0.258	0.222	5.557	0.013
5	FRic	functional	3	62.8	4.4	0.241	0.204	-6.032	0.017
6	Feeding type	1 function	3	63.1	4.7	0.233	0.196	-0.642	0.020
7	PD	phylogenetic	3	65.6	7.2	0.143	0.102	-0.002	0.075
8	FDiv_ab_	functional	3	65.8	7.4	0.136	0.094	-3.200	0.084
9	Env.*	other	8	66.1	7.7	0.432	0.219	-	0.121
10	Body length	1 function	3	66.3	7.9	0.118	0.076	-1.705	0.109
11	FEve_pa_	functional	3	66.3	7.9	0.115	0.073	-8.791	0.113
12	FDiv_pa_	functional	3	67.1	8.7	0.085	0.041	-10.069	0.177
13	SR	taxonomic	3	67.4	9.0	0.074	0.029	-0.581	0.211
14	Trophic group	1 function	3	67.4	9.0	0.073	0.029	-0.408	0.212
15	Zoop. Biomass	other	3	67.5	9.1	0.070	0.026	-0.875	0.221
16	Simpson	taxonomic	3	67.9	9.5	0.051	0.006	4.446	0.298
17	Shannon	taxonomic	3	68.2	9.8	0.041	-0.005	3.125	0.354
18	sesMPD_ab_	phylogenetic	3	68.3	9.9	0.034	-0.010	-0.323	0.388
19	Habitat type	1 function	3	68.5	10.1	0.027	-0.019	-0.469	0.451
20	TP	other	3	69.1	10.7	0.004	-0.044	-0.075	0.781
21	Env. PCA**	other	4	70.6	12.2	0.023	-0.075	-	0.796

The highest ranked model of each diversity type is bolded. *P* values that are less than 0.05 are bolded.

* Environmental variable model includes % tree cover, DIC, ln area, ln depth, pH, and ln DOC

** Environmental PCA includes first 2 axes of PCA on all standardized environmental variables.

The functional diversity measure with the lowest AIC was abundance weighted functional evenness (FEve_ab_; R_2_ = 0.26, *p* = 0.013), which in contrast to all other diversity measures, had a positive relationship with chlorophyll *a*. Again, excluding the outlying pond did not change the slope of the relationship but improved the model fit (R^2^ = 0.33, *p* = 0.005). The next best performing functional diversity measure was FRic (Fig. 3b; R^2^ = 0.24; *p* = 0.017). Again, excluding the outlying pond did not change the slope of the relationship but improved the model fit (Fig. 3b, dashed line; R^2^ = 0.44, *p* <0.001). The best measure of taxonomic diversity was species richness (SR; Fig. 3a; R^2^ = 0.07; *p* = 0.211), but no taxonomic diversity measure was able to explain a significant portion of variance in chlorophyll *a*. Again, excluding the outlying pond did not change the slope of the relationship but improved the model fit (Fig. 3a, dashed line; R^2^ = 0.17, *p* = 0.069).

Based on variation partitioning, SR, FRic, and sesMPD_pa_ together explained 29% of the variation in chlorophyll *a* (Fig. 3d). However, all variation explained was captured by sesMPD_pa_, either alone (4%) or with SR (8%) or FRic (25%). SR and FDiv each uniquely did not contribute to explaining variation in chlorophyll *a*, nor did the overlap between all three indices, and this resulted in less variation explained by the three indices together than that explained by sesMPD_pa_ on its own, because adjusted R^2^ penalizes for the additional degrees of freedom used in the combined model.

Two of the five traits (raptorial vs. filter feeding, and feeding type) individually explained a significant amount of variance in chlorophyll *a* and the raptorial vs. feeding type trait outperformed the best functional diversity measure (FRic)(Table 2). No single trait performed as well as the best phylogenetic diversity measure (sesMPD_pa_).

The best performing model for chlorophyll *a* containing only environmental variables consisted of % tree cover, DIC, ln pond area, ln depth, pH, and ln DOC but was not significant (*p* = 0.121). The model combining these environmental variables plus sesMPD_pa_ did not perform as well as the model with sesMPD_pa_ alone (Table 2). Neither zooplankton community biomass nor *D*. *pulex* biomass explained a significant amount of variation in chlorophyll *a* (Community Biomass R^2^ = 0.07; *p* = 0.221; *D*. *pulex*—R^2^ = 0.07; *p* = 0.221).

The pathway between sesMPD_pa_ and chlorophyll *a*, was always significant, regardless of how we specified the effect of environment in our SEM (S7 Fig.). Matching to the SEM with zooplankton community biomass, the most parsimonious model explaining variation in chlorophyll *a* did not include environment, but it included the direct pathway from sesMPD_pa_ (S2 Table). This again suggests that our linear models adequately capture the relationship between diversity and chlorophyll *a*.

## Discussion

Previous tests of the effect of functional and phylogenetic diversity on ecosystem functioning have been largely based on experimental plant communities [9,12,45]. Here, we evaluated these relationships across trophic levels in natural pond communities. Our linear models revealed strong and highly significant correlations between the functioning and the diversity of pond zooplankton communities, and our structural equation models demonstrated that these relationships were not simply driven by variation in environmental conditions. Both zooplankton functions considered here—the production of zooplankton biomass and top-down control of phytoplankton abundance—increased with diversity. This is consistent with previous experimental evidence and theory [16,18], but we found a clear relationship in complex and ephemeral natural communities, despite large variation in abiotic environments between ponds (e.g. phosphorous spans the natural gradient from oligotrophic 4.4 μg L^-1^ to hypereutrophic 315 μg L^-1^ [46]).

As predicted, we found that positive diversity ecosystem function relationships emerged most clearly when measures of functional and phylogenetic diversity were used, and that these measures explained variation in ecosystem function beyond that explained by taxonomic diversity measures, such as species richness. Previous studies relating the diversity of animals to ecosystem function have relied on taxonomic diversity measures [47,48], knowledge of the functional complementarity of species [49,50], single traits [13] or on taxonomic differences [16], but see [14]. Although we found a subset of single traits (e.g. trophic group, raptorial vs. filter feeding, and body length) performed almost as well in predicting zooplankton biomass, they never explained as much variation as the best diversity measures (FDiv_ab_). In addition, species richness and phylogenetic diversity (sesMPD_ab_) each explained additional unique variation. In contrast, phylogenetic diversity (sesMPD_pa_) was the best predictor of phytoplankton consumption, and no additional variation was uniquely explained by species richness and functional diversity (FRic). These findings suggest that metrics that quantitatively integrate trait or phylogenetic information have the potential to improve our understanding of variation in the functioning of complex multi-trophic ecosystems.

While species richness was a good predictor of zooplankton community biomass, the relationship was unimodal, and not a saturating function, as observed in most biodiversity-function research [2,18]. A thorough sampling of these ponds may have revealed additional rare species, although our rarefaction curves indicate that we sampled adequately to capture species richness in the majority of ponds. Nevertheless, our findings should be robust to this variability, as levels of both ecosystem functions were greatest with high functional and phylogenetic diversity, but not species richness. The unimodal relationship between species richness and zooplankton biomass is reminiscent of the commonly observed relationship between productivity and species richness at small spatial scales [51]. However, we find no evidence that productivity underlies the relationship described here, as zooplankton species richness was unrelated to either phytoplankton abundance or total phosphorous. The linear relationship between ecosystem function and both functional and phylogenetic diversity suggests that these measures better capture the diet complementarity between zooplankton species. It is not clear why functioning decreases at higher richness, but perhaps reflects an increasing representation of rare species that contribute little to ecosystem function, as we discuss further below.

The exception to the positive diversity ecosystem function relationship was the negative correlation between functional evenness and top-down control of phytoplankton. In this instance, functional evenness poorly reflects the complementarity of the zooplankton grazing function. Evenness is unaffected by the number of traits present within a community, rather, it is highest when the tips on the functional dendrogram of the community are evenly spaced [28]. For example, our community with the highest functional evenness was comprised entirely of copepods, which all are evenly spaced across the branch containing the raptorial feeders. However, no filter feeders were present within the community and so grazing complementarity was low. This highlights the need for careful consideration when choosing between indices. In contrast, functional richness provided a much more realistic estimation of grazing complementarity, and exhibited the predicted diversity function relationship.

Neither functional nor phylogenetic diversity were consistently the best predictor of ecosystem function, and the degree to which they explained unique variation differed depending on the measured function. While functional diversity (FDiv_ab_) explained the most variation in zooplankton biomass, both species richness and phylogenetic diversity (sesMPD_ab_) explained some overlapping and unique variation. In contrast, phylogenetic diversity (sesMPD_pa_) explained the most variation in phytoplankton abundance and although both species richness and functional diversity (FRic) explained overlapping variation, these were subsets of that explained by phylogenetic diversity. These findings suggest that the three types of diversity indices capture some of the same functional differences in community composition. This is perhaps unsurprising because functional traits and niche differences are often phylogenetically conserved [10,52], as reflected by the high overall correlation between the traits and phylogeny. However, the diversity measures did not overlap completely in the variance in ecosystem function that they explained, and each function was best predicted by a different diversity measure. For example, body length showed little phylogenetic signal, but was predictive of zooplankton community biomass, and this correlation may explain why functional diversity explained more variation than phylogenetic diversity for this ecosystem function. In contrast, the fact that phylogenetic diversity explained additional variation in phytoplankton abundance to that explained by functional traits is suggestive of other important, but unmeasured, functional differences that covary with phylogeny. Each class of metric thus captured some unique aspect of the way that the communities use resources [53], highlighting the value of combining different diversity metrics in models explaining ecosystem function.

The two aspects of zooplankton function we measured appear to be dependent on different aspects of community diversity. We intentionally chose diversity metrics that captured different aspects of community composition to provide insight into the mechanisms behind the diversity-ecosystem function relationships [28]. Thus we would not expect all of our diversity measures to correlate significantly with a given function, and it is this variation in predictive ability that provides insights into the different ways in which these communities exploit resources. This is highlighted by the fact that the two functions were best predicted by different subsets of our diversity indices; zooplankton community biomass was best predicted by abundance weighted measures of diversity, while the ability of this community to suppress phytoplankton through grazing was best predicted by diversity measures that only account for presence/absence. Furthermore, we found that there was no significant relationship between the two types of functions. This is surprising because we might have expected that the communities with the greatest biomass would be the most effective at grazing phytoplankton [54]. However, experimental evidence suggests that different functions are often produced by different subsets of the community [55], and our results support this interpretation.

The greatest zooplankton community biomass occurred in communities where abundance was spread between taxa that are functionally and phylogenetically distant. This was likely driven by the two most abundant taxa, *Daphnia pulex* and *Microcyclops rubellus*, which combined made up over two thirds of the average biomass in the ponds, but have very different functional traits, and are phylogenetically distant. The traits for trophic group, raptorial vs. filter feeding, and body length, were all predictive of total zooplankton biomass, and these taxa differ in all three of these traits. In contrast, both species share habitat preferences (pelagic habitats), and habitat preference diversity was not predictive of zooplankton community biomass. The disproportionate abundance of these two taxa resulted in a trade-off between species richness and functional or phylogenetic diversity so that the communities with the highest biomass generally had intermediate species richness. This decline in biomass with increasing species richness is due to the high number of rare taxa that contribute relatively little biomass to the community, which tend to be present when *D*. *pulex* is not abundant. We suspect that this negative correlation with *D*. *pulex* may be the result of competition, but it could also be due to factors such as differences in environmental preference. However, the relationship between community biomass and functional and phylogenetic diversity remained linear because the highest abundance-weighted diversity did not correspond to the highest species richness; the addition of rare taxa resulted in a small increase in abundance weighted diversity but this was more than compensated for by the corresponding reduction in *D*. *pulex* abundance.

Contrasting with the determinants of zooplankton community biomass, the ability for the zooplankton community to graze phytoplankton was dependent upon the presence of phylogenetically and functionally diverse taxa, regardless of abundances. We suggest that this is due to complementary grazing, whereby taxa specialize on different habitats and types of phytoplankton, and so communities with higher diversity were better able to suppress the abundance of all phytoplankton types. These functional differences appear to be well captured by our traits describing feeding type. For example, different cladoceran subgroups each employ a different type of filter feeding, while these copepod taxa are raptorial feeders. Similarly, Ye et al. [13] found that that the strength of top down control increases with the size diversity of marine zooplankton. Rare species have been found to contribute disproportionately to ecosystem functioning in communities of alpine plants, tropical trees, and coral fishes [56]. Given that abundances were not important for predicting top-down control in our ponds suggests this may also be the case for phytoplankton grazing by zooplankton.

Functional and phylogenetic diversity have been found to be informative of ecosystem functioning across trophic levels in a few other studies. Dinnage et al. [17] found a bottom-up effect of the phylogenetic diversity of plants on the diversity and abundance of arthropod herbivores and predators. In contrast, functional diversity, but not phylogenetic diversity, was informative of the grazing pressure of marine amphipods [14], highlighting the fact that the traits of interest may not always correlate with phylogeny. In our case, grazing appears to have been well captured by phylogenetic diversity, providing evidence of the value of these diversity measures for understanding ecosystem function across trophic levels.

Our diversity measures performed better than any combination of the local environmental variables in predicting both ecosystem functions. This includes total phosphorous, the limiting nutrient for phytoplankton growth in the vast majority of freshwater systems [57], but which was not retained as a significant predictor of phytoplankton abundance in our model. However, nitrogen and nitrogen-phosphorous co-limitation can also limit phytoplankton growth [58]. As we did not measure nitrogen concentrations, we cannot rule out that nutrient limitation may still play a role in these ponds. Nonetheless, our findings suggest that compositional differences in these zooplankton communities, which are captured in functional and phylogenetic diversity measures, have a larger impact on ecosystem function than do the local environmental conditions. This is supported by the results of our structural equation models, where our diversity measures were always the most significant predictors of ecosystem function, even when including environmental predictors. Furthermore, our most parsimonious model for predicting both ecosystem functions included only the relationship with zooplankton diversity.

## Conclusions

Our study provides evidence that the functional and phylogenetic diversity of natural zooplankton communities determines their ability to produce biomass, as well as suppress phytoplankton through top-down grazing. There is a good theoretical basis for the expectation that trait based functional and phylogenetic diversity measures should outperform simple taxonomic measures in explaining ecosystem function. However, previous use of these indices has been largely confined to experimental plant communities. Our study suggests that these indices can also increase our understanding of the functioning of ecosystems in natural environments. We suggest that the congruence of our results with clear a priori predictions based on a well-established body of theory and experimental evidence [10,16] provides support for our conclusions.

Furthermore, the two functions we explored here, biomass production and top-down control of phytoplankton, were each explained by different, but related, biodiversity metrics. These metrics provide insight into the underlying ecological processes responsible for each function. Zooplankton biomass production is best explained by functional diversity, whereas suppression of phytoplankton production was best explained by phylogenetic diversity. Therefore, we suggest combining functional and phylogenetic diversity measures to provide a richer understanding of the effects of biodiversity on ecosystem function.

## Supporting Information

S1 Fig The location of the 23 ponds on Mont St. Hilaire. The red outline marks the Gault Nature Reserve border. Map courtesy of Gault Nature Reserve.
(TIFF)

S2 Fig Dendrograms indicating the functional (a) and phylogenetic (b) relationships between the zooplankton in the Gault Nature Reserve ponds. All five traits were used to create the functional dendrogram (a) and we have marked traits that divide clearly across the main functional bifurcations. The four functional groups selected from the trait dendrogram (a) are distinguished by shade and these are retained in the phylogenetic tree (b).
(PDF)

S3 Fig The hypothesized paths by which zooplankton diversity and environmental factors could affect the two ecosystem functions. Note: because the link between phytoplankton abundance and zooplankton biomass is a trophic link, the direction of the arrow between these variables changes depending on which one we are trying to predict.
(PNG)

S4 Fig PCA of the 9 environmental variables. The black dots mark the position of the ponds in multivariate environmental space. All variables were standardized to a mean of zero and a standard deviation of one prior to calculating the PCA.
(TIFF)

S5 Fig Rarefaction curves estimating the relationship between species richness and the number of individuals identified in a sample for the 23 ponds. (TIFF)

S6 Fig Structural equation model to predict zooplankton biomass. This model is not the most parsimonious, but is shown because it includes all parameter types (zooplankton biomass, diversity, chlorophyll *a*, and environmental variables). Significant paths (*p < 0.05, **p < 0.01, ***p < 0.001) and their unstandardized parameter estimations are shown in black. Non-significant paths are shown in grey. Epsilons indicate error in endogenous variables. This diagram demonstrates that diversity was the most significant predictor of zooplankton biomass and was retained as significant when pathways from the environmental variables were included, as was the case in all models.
(TIFF)

S7 Fig Structural equation model to predict chlorophyll *a*. This model is not the most parsimonious, but is shown because it includes all parameter types (chlorophyll *a*, zooplankton biomass, diversity, and environmental variables). Significant paths (*p < 0.05, **p < 0.01, ***p < 0.001) and their unstandardized parameter estimations are shown in black. Epsilons indicate error in endogenous variables. Non-significant paths are shown in grey. This diagram demonstrates that diversity was the most significant predictor of chlorophyll *a* and was retained as significant when pathways from the environmental variables were included, as was the case in all models.
(TIFF)

S1 Table Structural equation models for predicting zooplankton community biomass ranked in increasing order of AIC. Zooplankton community biomass (Z.bmass) and chlorophyll *a* (chl) were ln transformed. The environmental variables selected through multiple regression (Env) were elevation, DIC, and log TP. PCA refers to the first two axes of a PCA of all standardized environmental variables. The χ^2^ test provides a test of how well the model fits the data. Models with *p*-values >0.05 are considered to be a reasonable fit to the data. Models are saturated when paths are specified between all variables and are considered to fit the data perfectly (Grace 2006).
(DOCX)

S2 Table Structural equation models for predicting chlorophyll *a* ranked in increasing order of AIC. Zooplankton community biomass (Z.bmass), chlorophyll *a* (chl), and total phosphorous (TP) were ln transformed. The environmental variables selected through multiple regression (Env) were % tree cover, DIC, log area, log depth, pH, and log DOC. PCA refers to the first two axes of a PCA of all standardized environmental variables. The χ^2^ test provides a test of how well the model fits the data. Models with *p*-values >0.05 are considered to be a reasonable fit to the data. Models are saturated when paths are specified between all variables and are considered to fit the data perfectly (Grace 2006).
(DOCX)

S1 File Supporting data. Zooplankton, chlorophyll *a*, environmental, spatial, trait, and phylogeny data for all ponds and species.
(XLS)

## References

[1] Hooper DU, Chapin FS, Ewel JJ, Hector A, Inchausti P, et al (2005) Effects of biodiversity on ecosystem functioning: a consensus of current knowledge. Ecol Monogr 75: 3–35. 10.1890/04-0922

[2] Cardinale BJ, Matulich KL, Hooper DU, Byrnes JE, Duffy E, et al (2011) The functional role of producer diversity in ecosystems. Am J Bot 98: 572–592. 10.3732/ajb.1000364 21613148

[3] Cardinale BJ, Srivastava DS, Duffy JE, Wright JP, Downing AL, et al (2006) Effects of biodiversity on the functioning of trophic groups and ecosystems. Nature 443: 989–992. 10.1038/nature05202 17066035

[4] Reiss J, Bridle JR, Montoya JM, Woodward G (2009) Emerging horizons in biodiversity and ecosystem functioning research. Trends Ecol Evol 24: 505–514. 10.1016/j.tree.2009.03.018 19595476

[5] Cadotte MW, Carscadden K, Mirotchnick N (2011) Beyond species: functional diversity and the maintenance of ecological processes and services. J Appl Ecol 48: 1079–1087. 10.1111/j.1365-2664.2011.02048.x

[6] Naeem S, Duffy JE, Zavaleta E (2012) The functions of biological diversity in an age of extinction. Science 336: 1401–1406. 10.1126/science.1215855 22700920

[7] Petchey OL, Gaston KJ (2006) Functional diversity: back to basics and looking forward. Ecol Lett 9: 741–758. 10.1111/j.1461-0248.2006.00924.x 16706917

[8] Petchey OL, Hector A, Gaston KJ (2004) How Do Different Measures of Functional Diversity Perform? Ecology 85: 847–857. 10.1890/03-0226

[9] Flynn DFB, Mirotchnick N, Jain M, Palmer MI, Naeem S (2011) Functional and phylogenetic diversity as predictors of biodiversity—ecosystem-function relationships. Ecology 92: 1573–1581. 10.1890/10-1245.1 21905424

[10] Srivastava DS, Cadotte MW, MacDonald AAM, Marushia RG, Mirotchnick N (2012) Phylogenetic diversity and the functioning of ecosystems. Ecol Lett 15: 637–648. 10.1111/j.1461-0248.2012.01795.x 22583836

[11] Cadotte MW, Cavender-Bares J, Tilman D, Oakley TH (2009) Using Phylogenetic, Functional and Trait Diversity to Understand Patterns of Plant Community Productivity. PLoS ONE 4: e5695 10.1371/journal.pone.0005695 19479086 PMC2682649

[12] Clark CM, Flynn DFB, Butterfield BJ, Reich PB (2012) Testing the Link between Functional Diversity and Ecosystem Functioning in a Minnesota Grassland Experiment. PLoS ONE 7: e52821 10.1371/journal.pone.0052821 23300787 PMC3534119

[13] Ye L, Chang C-Y, García-Comas C, Gong G-C, Hsieh C-H (2013) Increasing zooplankton size diversity enhances the strength of top-down control on phytoplankton through diet niche partitioning. J Anim Ecol 82: 1052–1061. 10.1111/1365-2656.12067 23506226

[14] Best RJ, Caulk NC, Stachowicz JJ (2012) Trait vs. phylogenetic diversity as predictors of competition and community composition in herbivorous marine amphipods. Ecol Lett 16: 72–80. 10.1111/ele.12016 23066869

[15] Leduc D, Rowden AA, Pilditch CA, Maas EW, Probert PK (2013) Is there a link between deep-sea biodiversity and ecosystem function? Mar Ecol Prog Ser 34: 334–344. 10.1111/maec.12019

[16] Griffin JN, Byrnes J, Cardinale BJ (2013) Effects of predator richness on prey suppression: a meta-analysis. Ecology 94: 2180–2187. 10.1890/13-0179.1 24358704

[17] Dinnage R, Cadotte MW, Haddad NM, Crutsinger GM, Tilman D (2012) Diversity of plant evolutionary lineages promotes arthropod diversity. Ecol Lett 15: 1308–1317. 10.1111/j.1461-0248.2012.01854.x 22913753

[18] Duffy JE, Cardinale BJ, France KE, McIntyre PB, Thébault E, et al (2007) The functional role of biodiversity in ecosystems: incorporating trophic complexity. Ecol Lett 10: 522–538. 10.1111/j.1461-0248.2007.01037.x 17498151

[19] McQueen DJ, Post JR, Mills EL (1986) Trophic relationships in freshwater pelagic ecosystems. Can J Fish Aquat Sci 43: 1571–1581.

[20] Cardinale BJ, Bennett DM, Nelson CE, Gross K (2009) Does productivity drive diversity or vice versa? A test of the multivariate productivity-diversity hypothesis in streams. Ecology 90: 1227–1241. 19537544 10.1890/08-1038.1

[21] Haney JF (2010) An-image-based Key to the Zooplankton of the Northeast, USA. version 5.0 released 2013. University of New Hampshire Center for Freshwater Biology

[22] McCauley E (1984) The estimation of the abundance and biomass of zooplankton in samples In: Downing J, Rigler FH, editors. A Manual on Methods for the Assessment of Secondary Productivity in Fresh Waters Oxford, UK: Blackwell Scientific pp. 228–265.

[23] Wetzel RG, Likens GE (1979) Limnological Analyses . New York, NY: Springer

[24] Oksanen J, Blanchet FG, Kindt R, Legendre P, Minchin PR, et al. (2011) vegan: Community Ecology Package. R package version 2.0–10. Available: http://CRAN.R-project.org/package=vegan.

[25] R Development Core Team (2014) R: A language and environment for statistical computing. Version 3.0.2. Available: http://www.R-project.org/.

[26] Petchey OL, O’Gorman E, Flynn DFB (2009) A functional guide to functional diversity measures In: Naeem S, Bunker D, Hector A, Loreau M, Perrings C, editors. Biodiversity, Ecosystem Functioning, and Human Wellbeing. Oxford: Oxford University Press 10.1093/acprof:oso/9780199547951.003.0004

[27] Cadotte MW, Jonathan Davies T, Regetz J, Kembel SW, Cleland E, et al (2010) Phylogenetic diversity metrics for ecological communities: integrating species richness, abundance and evolutionary history. Ecol Lett 13: 96–105. 10.1111/j.1461-0248.2009.01405.x 19903196

[28] Villeger S, Mason NW, Mouillot D (2008) New multidimensional functional diversity indices for a multifaceted framework in functional ecology. Ecology 89: 2290–2301. 10.1890/07-1206.1 18724739

[29] Barnett AJ, Finlay K, Beisner BE (2007) Functional diversity of crustacean zooplankton communities: towards a trait-based classification. Freshwater Biol 52: 796–813. 10.1111/j.1365-2427.2007.01733.x

[30] Laliberté E, Legendre P, Shipley B (2014) FD: measuring functional diversity from multiple traits, and other tools for functional ecology. R package version. 1.0–12. 10.1890/08-2244.1 20380219

[31] Faith DP (1992) Conservation evaluation and phylogenetic diversity. Biol Conserv 61: 1–10. 10.1016/0006-3207(92)91201-3

[32] Webb CO, Ackerly DD, McPeek MA, Donoghue MJ (2002) Phylogenies and Community Ecology. Annu Rev Ecol Syst 33: 475–505. 10.1146/annurev.ecolsys.33.010802.150448

[33] Kembel SW, Cowan PD, Helmus MR, Cornwell WK, Morlon H, et al (2010) Picante: R tools for integrating phylogenies and ecology. Bioinformatics 26: 1463–1464. 10.1093/bioinformatics/btq166 20395285

[34] Helmus MR, Keller WB, Paterson MJ, Yan ND, Cannon CH, et al (2010) Communities contain closely related species during ecosystem disturbance. Ecol Lett 13: 162–174. 10.1111/j.1461-0248.2009.01411.x 20015255

[35] Huys R, Boxshall GA (1991) Copepod Evolution. London: The Ray Society.

[36] Legendre P (2013) lmodel2: Model II Regression. R package version 1.7–2. Available: http://CRAN.R-project.org/package=lmodel2.

[37] Pavoine S, Gasc A, Bonsall MB, Mason NWH (2013) Correlations between phylogenetic and functional diversity: mathematical artefacts or true ecological and evolutionary processes? J Veg Sci 24: 781–793. 10.1111/jvs.12051

[38] Fritz SA, Purvis A (2010) Selectivity in mammalian extinction risk and threat types: a new measure of phylogenetic signal strength in binary traits. Conserv Biol 24: 1042–1051. 10.1111/j.1523-1739.2010.01455.x 20184650

[39] Orme D, Freckleton R, Thomas G, Petzoldt T, Fritz SA, et al. (2013) caper: Comparative Analyses of Phylogenetics and Evolution in R. R package version 0.5.2. Available: http://CRAN.R-project.org/package=caper.

[40] Pagel M (1999) Inferring the historical patterns of biological evolution. Nature 401: 877–884. 10553904 10.1038/44766

[41] Harmon LJ, Weir JT, Brock CD, Glor RE, Challenger W (2008) GEIGER: investigating evolutionary radiations. Bioinformatics 24: 129–131. 18006550 10.1093/bioinformatics/btm538

[42] Grace JB (2006) Structural Equation Modeling and Natural Systems. Cambridge: Cambridge University Press.

[43] Lumley T, Miller A (2009) leaps: regression subset selection. R package 2.9. Available: http://CRAN.R-project.org/package=leaps

[44] Brandl Z (1998) Feeding strategies of planktonic cyclopoids in lacustrine ecosystems. J Marine Syst 15: 87–95. 9523213

[45] Cadotte MW (2013) Experimental evidence that evolutionarily diverse assemblages result in higher productivity. P Natl Acad Sci USA 110: 8996–9000. 10.1073/pnas.1301685110 23674676 PMC3670319

[46] Carlson RE (1977) A trophic state index for lakes. Limnol Oceanogr 22: 361–369.

[47] Gamfeldt L, Hillebrand H, Jonsson PR (2005) Species richness changes across two trophic levels simultaneously affect prey and consumer biomass. Ecol Lett 8: 696–703. 10.1111/j.1461-0248.2005.00765.x

[48] Petchey OL, McPhearson PT, Casey TM, Morin PJ (1999) Environmental warming alters food-web structure and ecosystem function. Nature 402: 69–72. 10.1038/47023

[49] Burkepile DE, Hay ME (2008) Herbivore species richness and feeding complementarity affect community structure and function on a coral reef. P Natl Acad Sci USA 105: 16201–16206. 10.1073/pnas.0801946105 18845686 PMC2565647

[50] Schmitz OJ (2009) Effects of predator functional diversity on grassland ecosystem function. Ecology 90: 2339–2345. 10.1890/08-1919.1 19769111

[51] Mittelbach GG, Steiner CF, Scheiner SM, Gross KL, Reynolds HL, et al (2001) What is the observed relationship between species richness and productivity? Ecology 82: 2381–2396.

[52] Freckleton RP, Harvey PH, Pagel M (2002) Phylogenetic Analysis and Comparative Data: A Test and Review of Evidence. Am Nat 160: 712–726. 10.1086/343873 18707460

[53] Loreau M, Hector A (2001) Partitioning selection and complementarity in biodiversity experiments. Nature 412: 72–76. 10.1038/35083573 11452308

[54] Jeppesen E, Jensen JP, Spndergaard M, Torben Lauridsen LJPLJ (1997) Top-down control in freshwater lakes: the role of nutrient state, submerged macrophytes and water depth. Hydrobiologia 342/343: 151–164.

[55] Zavaleta ES, Pasari JR, Hulvey KB, Tilman GD (2010) Sustaining multiple ecosystem functions in grassland communities requires higher biodiversity. P Natl Acad Sci USA 107: 1443–1446. 10.1073/pnas.0906829107 20080690 PMC2824370

[56] Mouillot D, Bellwood DR, Baraloto C, Chave J, Galzin R, et al (2013) Rare Species Support Vulnerable Functions in High-Diversity Ecosystems. PLoS Biol 11: e1001569 10.1371/journal.pbio.1001569 23723735 PMC3665844

[57] Schindler DW (1977) Evolution of Phosphorus Limitation in Lakes. Science 195: 260–262. 10.1126/science.195.4275.260 17787798

[58] Elser JJ, Marzolf ER, Goldrnan CR (1990) Phosphorous and Nitrogen Limitation of Phytoplankton Growth in the Freshwaters of North America: A Review and Critique of Experimental Enrichments. Can J Fish Aquat Sci 47: 1468–1477.

