# Correction: Ecosystem Functions across Trophic Levels Are Linked to Functional and Phylogenetic Diversity

**DOI:** 10.1371/journal.pone.0220213

**Published:** 2019-07-18

**Authors:** Patrick L. Thompson, T. Jonathan Davies, Andrew Gonzalez

The authors have discovered that an indexing error occurred in the code for the analysis of the article. Despite this error, the general conclusions of the article remain supported by the data: ecosystem functions across trophic levels are linked to functional and phylogenetic diversity. Furthermore, phylogenetic and functional diversity measures explain variation in ecosystem functions beyond that which is explained by traditional diversity measures such as species richness. After rerunning analysis, functional diversity is no longer the best predictor of zooplankton biomass. Rather, functional diversity, together with phylogenetic diversity, explains all the variation explained by species richness, along with additional unique variation. All together, this indexing error resulted in errors in Figs 2 and 3 and S6 and S7 and Tables 1 and 2 and S1 and S2 and S1 File, as well as in the Abstract, Results, Discussion, and Conclusion sections.

Please see the corrected Figs 2 and 3 and Tables 1 and 2 here; please view the corrected S6 and S7 Figs and S1 and S2 Tables and S1 File below.

**Fig 2:**
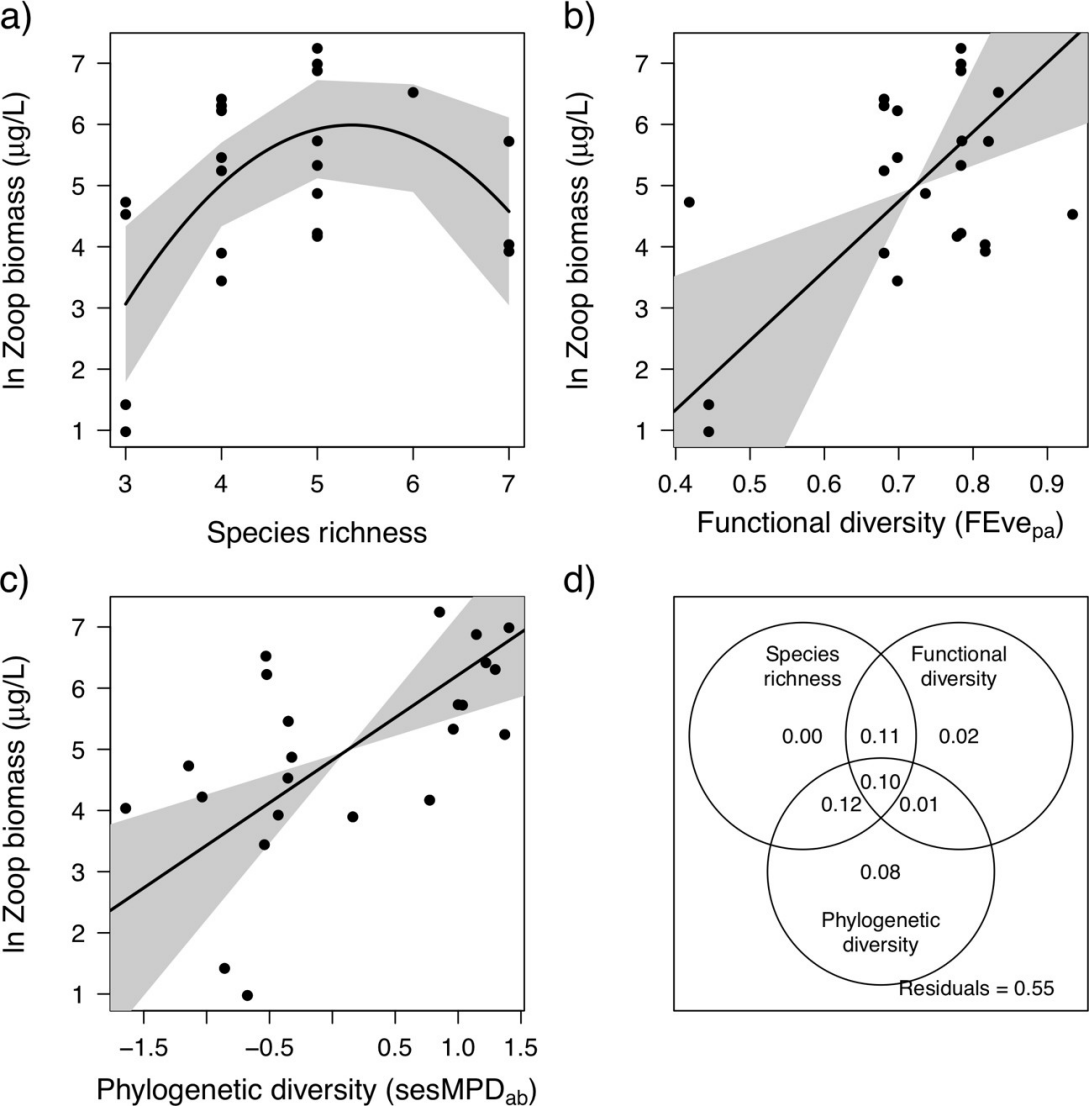
**Zooplankton community biomass in the 23 ponds as predicted by the best diversity indices in each category: taxonomic diversity—species richness (a), functional diversity—abundance weighted functional divergence (b), phylogenetic diversity—abundance weighted standard effect size mean pairwise distance (c), and the variation partitioning for these three models with their adjusted R**^**2**^ **(d).** Significant model trends are shown as black lines. The grey bands indicate the 95% confidence intervals for the predicted values (a) and the slope of the regression lines (b,c).

**Fig 3:**
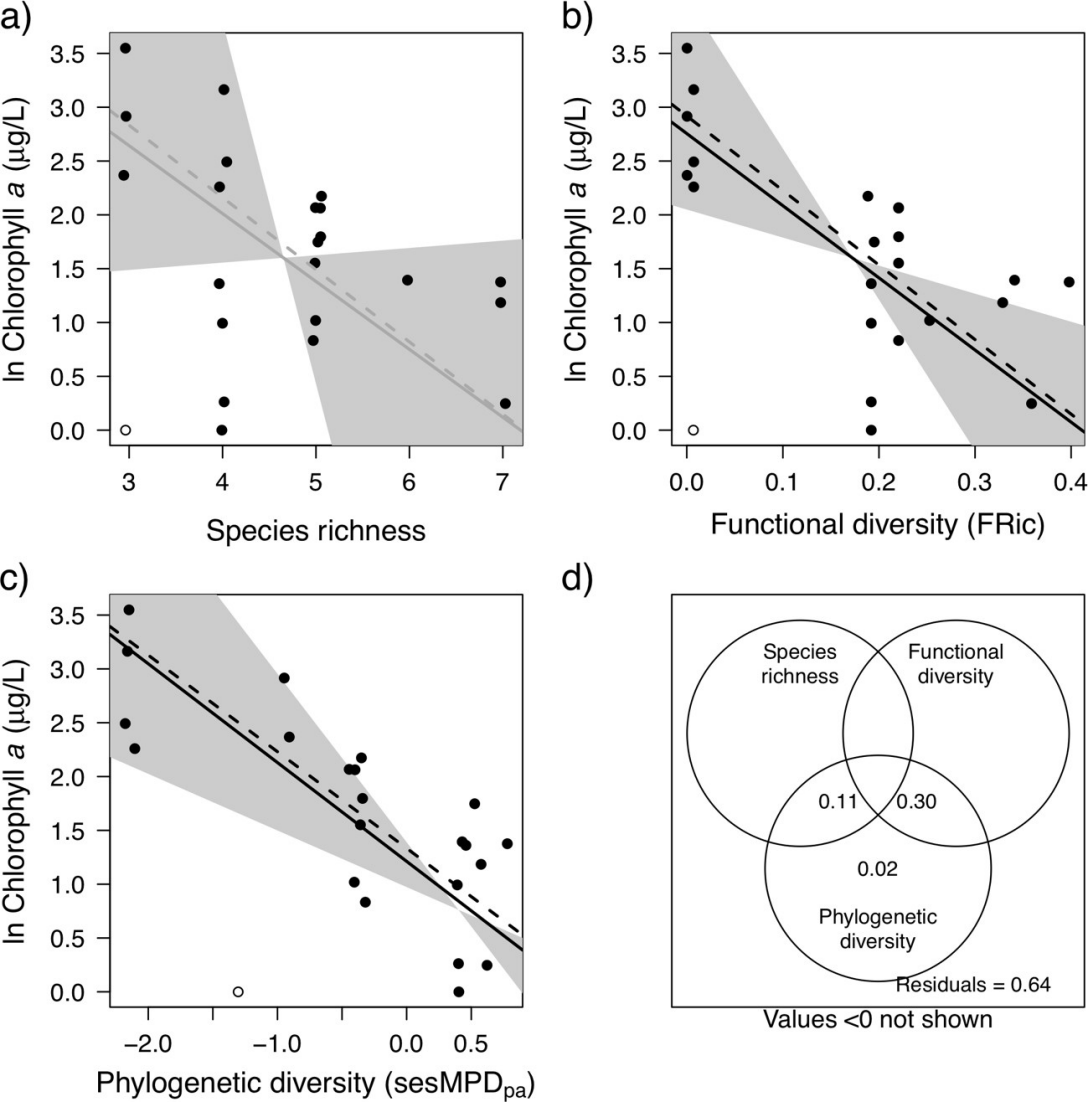
**Chlorophyll a in the 23 ponds as predicted by the best diversity indices in each category: taxonomic diversity—species richness (a), functional diversity–functional richness (b), phylogenetic diversity–presence absence standard effect size mean pairwise distance (c), and the variation partitioning for these three models with their adjusted R^2^ (d).** Significant model trends are shown as black lines. Insignificant model trends are shown as grey lines. The empty circles indicate the pond that is an outlier to the predicted trend. The dashed lines indicate the model trend when this outlier is removed. The grey bands indicate 95% confidence intervals for the slope of the regression lines.

**Table 1:** Results of the linear models for predicting zooplankton community biomass (ln), ranked in increasing order of AIC.

	variable	type	d.f.	AIC	ΔAIC	R^2^	R^2^ Adj	slope	*p*
1	sesMPD_ab_ + Env*	other	4	78.2	0.0	0.548	0.477	-	**0.001**
2	SR^2^ + Env*	other	5	78.8	0.6	0.575	0.481	-	**0.003**
3	Body size	1 trait	2	82.7	4.3	0.346	0.315	7.50	**0.003**
4	**SR**^**2**^	**taxonomic**	3	82.8	4.5	0.397	0.336	5.63*x -0.53x^2	**0.006**
5	**sesMPD**_**ab**_	**phylogenetic**	2	82.8	4.6	0.342	0.310	1.38	**0.003**
6	Env*	other	3	83.0	5.0	0.391	0.330	-	**0.007**
7	**FEve**_**pa**_	**functional**	2	85.1	6.9	0.274	0.239	11.35	**0.010**
8	Trophic group	1 trait	2	86.0	7.8	0.244	0.208	1.40	**0.017**
9	Raptorial vs. filter	1 trait	2	86.5	8.3	0.227	0.191	1.97	**0.021**
10	FRic	functional	2	88.8	10.6	0.145	0.105	7.75	0.073
11	PD	phylogenetic	2	88.9	10.7	0.145	0.104	2.11e-3	0.073
12	FDiv_pa_	functional	2	89.0	10.8	0.141	0.100	14.72	0.078
13	Feeding type	1 trait	2	90.1	11.9	0.099	0.056	1.02	0.143
14	SR	taxonomic	2	90.2	12.0	0.093	0.050	0.72	0.158
15	Simpson	taxonomic	2	90.2	12.0	0.092	0.049	-5.69	0.158
16	Env. PCA**	other	3	90.2	12.4	0.168	0.084	-	0.160
17	Shannon	taxonomic	2	91.0	12.8	0.060	0.015	-3.81	0.260
18	FDiv_ab_	functional	2	91.4	13.2	0.045	0.000	5.54	0.330
19	sesMPD_pa_	phylogenetic	2	91.4	13.4	0.045	-0.001	0.64	0.332
20	Chl *a*	other	2	92.0	13.8	0.018	-0.029	-0.60	0.542
21	FEve_ab_	functional	2	92.2	14.0	0.012	-0.035	-2.48	0.622
22	Habitat type	1 trait	2	92.3	14.1	0.006	-0.041	-0.64	0.718

The highest ranked model of each diversity type is bolded. *P* values that are less than 0.05 are bolded.

* Environmental variable model includes elevation and ln TP

** Environmental PCA includes first 2 axes of PCA on all standardized environmental variables

**Table 2:** Results of the linear models for predicting chlorophyll a (ln), ranked in increasing order of AIC.

	variable	type	d.f.	AIC	ΔAIC	R^2^	R^2^ Adj.	slope	*p*
1	**sesMPD**_**pa**_	**phylogenetic**	2	55.9	0.0	0.439	0.412	-0.94	**0.001**
2	sesMPDpa + Env*	other	7	56.2	0.3	0.631	0.492	-	**0.007**
3	FEve_pa_*	functional	2	59.6	3.7	0.338	0.307	-8.10	**0.004**
4	Raptorial vs. filter	1 trait	2	60.9	5.0	0.301	0.267	-1.44	**0.007**
5	**FRic**	**functional**	2	61.7	5.8	0.277	0.242	-6.70	**0.010**
6	Feeding type	1 trait	2	63.1	7.2	0.233	0.196	-0.64	**0.020**
7	Env.**	other	6	63.4	7.5	0.449	0.287	-1.00	0.052
8	PD	phylogenetic	2	64.0	8.1	0.200	0.162	-1.80e-3	**0.033**
9	Trophic group	1 trait	2	65.9	10.0	0.131	0.090	-0.81	0.089
10	Body length	1 trait	2	66.0	10.1	0.127	0.085	-5.65	0.095
11	**SR**	**taxonomic**	2	66.7	10.8	0.101	0.058	-0.63	0.140
12	sesMPD_ab_	phylogenetic	2	67.3	11.4	0.079	0.035	-0.80	0.195
13	Simpson	taxonomic	2	67.7	11.8	0.062	0.017	5.53	0.253
14	FDiv_ab_	functional	2	68.2	12.3	0.040	-0.006	-5.40	0.362
15	FDiv_pa_	functional	2	68.3	12.4	0.038	-0.008	-8.58	0.373
16	Shannon	taxonomic	2	68.5	12.6	0.029	-0.017	5.17	0.437
17	Habitat type	1 trait	2	68.5	12.6	0.027	-0.019	-1.16	0.451
18	Zoop. Biomass	other	2	68.7	12.8	0.018	-0.029	-0.89	0.542
19	FEve_ab_	functional	2	69.1	13.2	0.001	-0.047	-5.75	0.898
20	Env. PCA***	other	3	69.8	13.9	0.055	-0.039	-	0.565

The highest ranked model of each diversity type is bolded. *P* values that are less than 0.05 are bolded.

* FEve_pa_ had the lowest AIC of any functional diversity measure but this model fit was highly dependent on the pond with suspect outlier Chl *a* concentration. The second best functional diversity measure had a similar AIC but was not sensitive to this outlier so we have elected to select this as our best fit functional diversity measure.

** Environmental variable model includes ln depth, ln pH, ln DIC, ln Wet Days, and ln TP

*** Environmental PCA includes first 2 axes of PCA on all standardized environmental variables

Please see the corrected Abstract, Results, Discussion, and Conclusion text here.

In the Abstract, there is an error in the ninth sentence. This sentence should be replaced with the following two sentences:

“Measures of zooplankton phylogenetic diversity and trait-based functional diversity together explained all the variation in zooplankton community biomass that was explained by species richness, in addition to unique variation that was not explained by species richness alone. In contrast, phytoplankton abundance was best predicted by zooplankton phylogenetic diversity, which explained all variation explained by any of the three types of diversity indices.”

In the Zooplankton community characteristics subsection of the Results section, there are a number of errors in the first and second paragraph. The correct first and second paragraphs are:

“Average pond species richness was 4.65, ranging from 3 to 7, with a regional richness of 10. Across all ponds, zooplankton community biomass was 334.77 μg L^-1^ on average, ranging from 2.66 to 1398.85 μg L^-1^. Species richness was positively, albeit weakly, related to the number of zooplankton present in our samples (R^2^ = 0.29; *p* = 0.006). However, the rarefaction curves saturated in the majority of samples (87%), suggesting that our estimates of richness were not greatly biased by differences in the abundance of zooplankton amongst the ponds (Figure S5).

*Daphnia pulex* comprised 44.9% of the zooplankton biomass over all ponds and was present in 14 of the 23 ponds. The next most abundant taxon, *Harpactocoid* copepods, comprised 26.4% of the zooplankton biomass in all ponds and was present in 21 of the 23 ponds. *Acanthocyclops vernalis* comprised 10.6% of the zooplankton biomass in all ponds and was present in 15 of the 23 ponds. This species is carnivorous as an adult but was retained in our analysis because it consumes phytoplankton in its juvenile stages [44]. There were seven rarer taxa, *Alonella sp*., *Ceriodaphnia dubia*, *Chydorus sphaericus*, Harpacticoida, *Sida crystallina*, *Simocephalus sp*., and *Tropocyclops prasinus mexicanus*, that each made up less than 5% of the average biomass. Phytoplankton abundance was 6.56 μg chl *a* L^-1^ on average, ranging from undetectable to 33 μg L^-1^. Zooplankton species richness was not related to either chlorophyll *a* (R^2^ = 0.10; *p* = 0.140) or total phosphorous (R^2^ = 0.11; *p* = 0.131).”

In the Zooplankton community biomass subsection of the Results section, there are a number of errors. The correct Zooplankton community biomass subsection is:

“Three out of the 11 diversity measures tested explained a significant proportion of variance in zooplankton community biomass, and in all cases, there was a positive influence of diversity on biomass (Table 1). These significant models included taxonomic, functional, and phylogenetic measures. Species richness (SR; Fig 2A; R^2^ = 0.38; *p* = 0.008) and abundance weighted standard effect size mean pairwise distance (sesMPD_ab_; Fig 2C; R^2^ = 0.34; *p* = 0.003), were jointly selected (equal AIC) as the best single diversity measure predictors of zooplankton community biomass. Species richness (SR) exhibited a unimodal relationship with zooplankton community biomass, where the highest biomass was found in ponds with intermediate species richness. This unimodal relationship between species richness and community zooplankton biomass outperformed a model that assumed a linear relationship (R^2^ = 0.14, *p* = 0.077). Presence absence weighted functional evenness (Feve_pa_) explained the third highest proportion of variance of the single diversity measure models (Fig 2B; R^2^ = 0.27; *p* = 0.010).

Based on variation partitioning, SR, Feve_pa_, and sesMPD_ab_ together explained 45% of the variation in zooplankton community biomass, and overlapped in explaining 10% of the variation (Fig 2D). SR and Feve_pa_ overlapped to explain 11% of the variation. SR and sesMPD_ab_ overlapped to explain 12% of the variation. Feve_pa_ and sesMPD_ab_ overlapped to explain 1% of the variation. SR, Feve_pa_, and sesMPD_ab_ uniquely explained 0%, 2%, and 8% of the variation respectively.

Three of the five traits (trophic group, raptorial vs. filter feeding, and body length) individually explained a significant amount of variance in zooplankton biomass and body length outperformed the best functional diversity measures although ΔAIC was small (Table 1).

The best performing model of environmental variables for predicting zooplankton community biomass consisted of elevation and ln TP, and explained a significant amount of variation (R^2^ = 0.39; *p* = 0.007). However, when either sesMPD_ab_ or species richness was included in the model, none of these environmental variables remained as significant predictors, although the models outperformed all other models (Table 1). Chlorophyll *a* did not explain a significant amount of variance in zooplankton community biomass (R^2^ = 0.02; *p* = 0542).

The pathway between sesMPD_ab_ (chosen for use in the SEM models because it performed equally well to SR and was the best predictor when combined with the environmental variables) and zooplankton community biomass, was always significant, regardless of how we specified the effect of environment in our SEM (S6 Fig). The most parsimonious model, based on AIC, only included the direct pathway from sesMPD_ab_ (S1 Table). This suggests that our linear models adequately capture the relationship between diversity and zooplankton biomass.”

In the Phytoplankton abundance subsection of the Results section, there are a number of errors. The correct Phytoplankton abundance subsection is:

“Four out of the 11 zooplankton diversity measures explained a significant proportion of variance in chlorophyll *a*, and in all cases there was a negative influence of diversity on chlorophyll *a* (Table 2). These significant models included functional and phylogenetic, but not taxonomic diversity measures. The best single diversity measure for predicting chlorophyll *a* was the phylogenetic diversity measure sesMPD_pa_ (Fig 3C; R^2^ = 0.44; *p* = 0.001). There was one outlier in this relationship, which was found to have significant influence (Cook’s distance > 0.5) on the analysis (Fig 3C—unfilled point). Chlorophyll *a* was not detectable in this pond, although the predicted concentration should have been relatively high based on the measured zooplankton phylogenetic diversity. We cannot be sure if this chlorophyll *a* concentration is a measurement error, so we compared model fit with and without including it. Removing this outlier from our analysis did not have a large effect on the slope of the relationship but greatly improved the model fit (Fig 3C, dashed line; R^2^ = 0.63, *p* <0.001).

The functional diversity measure with the lowest AIC was abundance weighted functional evenness (FEve_pa_; R_2_ = 0.34, *p* = 0.004). However, this model slope was highly sensitive to the inclusion of the outlier (S7 Fig), and the next best functional diversity measure FRic had a similar AIC (Table 2) but was not as influenced by the outlier (Fig 3B) and so we have elected to use FRic as our best functional diversity measure. FRic had a positive relationship with chlorophyll *a* (Fig 3B; R_2_ = 0.28, *p* = 0.010) and excluding the outlying pond did not change the slope of the relationship but improved the model fit (R^2^ = 0.49, *p* < 0.001). The best measure of taxonomic diversity was species richness (SR; Fig 3A; R^2^ = 0.10; *p* = 0.140), but no taxonomic diversity measure was able to explain a significant portion of variance in chlorophyll *a*. Again, excluding the outlying pond did not change the slope of the relationship but improved the model fit (Fig 3A, dashed line; R^2^ = 0.22, *p* = 0.02).

Based on variation partitioning, SR, FRic, and sesMPD_pa_ together explained 37% of the variation in chlorophyll *a* (Fig 3D). However, all variation explained was captured by sesMPD_pa_, either alone (4%) or with SR (11%) or FRic (30%). SR and FDiv each uniquely did not contribute to explaining variation in chlorophyll *a*, nor did the overlap between all three indices, and this resulted in less variation explained by the three indices together than that explained by sesMPD_pa_ on its own, because adjusted R^2^ penalizes for the additional degrees of freedom used in the combined model.

Two of the five traits (raptorial vs. filter feeding, and feeding type) individually explained a significant amount of variance in chlorophyll *a* (Table 2). No single trait performed as well as the best phylogenetic diversity measure (sesMPD_pa_).

The best performing model for chlorophyll *a* containing only environmental variables consisted of ln DIC, ln depth, ln pH, ln wet days and ln TP but was not significant (*p* = 0.052). The model combining these environmental variables plus sesMPD_pa_ did not perform as well as the model with sesMPD_pa_ alone (Table 2). Neither zooplankton community biomass nor *D*. *pulex* biomass explained a significant amount of variation in chlorophyll *a* (Community Biomass R^2^ = 0.02; *p* = 0.542; *D*. *pulex*–R^2^ = 0.07; *p* = 0.221).

The pathway between sesMPD_pa_ and chlorophyll *a*, was always significant, regardless of how we specified the effect of environment in our SEM (Figure S8). Matching to the SEM with zooplankton community biomass, the most parsimonious model explaining variation in chlorophyll *a* did not include environment, but it included the direct pathway from sesMPD_pa_ (S2 Table). This again suggests that our linear models adequately capture the relationship between diversity and chlorophyll *a*.”

In the Discussion section, there are errors in the second, fourth, and fifth paragraph. The correct second paragraph is:

“As predicted, we found that positive diversity ecosystem function relationships emerged most clearly when measures of functional and phylogenetic diversity were used, and that these measures explained variation in ecosystem function beyond that explained by taxonomic diversity measures, such as species richness. Previous studies relating the diversity of animals to ecosystem function have relied on taxonomic diversity measures [47,48], knowledge of the functional complementarity of species [49,50], single traits [13] or on taxonomic differences [16], but see [14]. Species richness, phylogenetic diversity (sesMPD_ab_), and functional diversity (Feve_pa_) all explained significant amounts of variation in zooplankton biomass. Furthermore, none of this variation was explained solely by species richness, whereas phylogenetic diversity and functional diversity each explained additional unique variation. Although we found a subset of single traits (e.g. trophic group, raptorial vs. filter feeding, and body length) performed almost as well in predicting zooplankton biomass, only body size explained as much variation as the best diversity measures. In contrast, phylogenetic diversity (sesMPD_pa_) was the best predictor of phytoplankton consumption, and no additional variation was uniquely explained by species richness and functional diversity (FRic). These findings suggest that metrics that quantitatively integrate trait or phylogenetic information have the potential to improve our understanding of variation in the functioning of complex multi-trophic ecosystems.”

The fourth paragraph of the Discussion section is no longer relevant after the reanalysis and should be removed.

The correct fifth paragraph of the Discussion is:

“No single diversity index was consistently the best predictor of ecosystem function, and the degree to which they explained unique variation differed depending on the measured function. While taxonomic diversity (SR^2^) explained the most variation in zooplankton biomass, all of this variation was captured by either phylogenetic diversity (sesMPD_ab_) or functional diversity (Feve_pa_), which each captured additional unique variation. In contrast, phylogenetic diversity (sesMPD_pa_) explained the most variation in phytoplankton abundance and although both species richness and functional diversity (FRic) explained overlapping variation, these were subsets of that explained by phylogenetic diversity. These findings suggest that the three types of diversity indices capture some of the same functional differences in community composition. This is perhaps unsurprising because functional traits and niche differences are often phylogenetically conserved [10,52], as reflected by the high overall correlation between the traits and phylogeny. However, the diversity measures did not overlap completely in the variance in ecosystem function that they explained, and each function was best predicted by a different diversity measure. For example, body size showed little phylogenetic signal, but was predictive of zooplankton community biomass, and this correlation may explain why functional diversity explained unique variation not captured by phylogenetic diversity for this ecosystem function. In contrast, the fact that phylogenetic diversity explained additional variation in phytoplankton abundance to that explained by functional traits is suggestive of other important, but unmeasured, functional differences that covary with phylogeny. Each class of metric thus captured some unique aspect of the way that the communities use resources [53], highlighting the value of combining different diversity metrics in models explaining ecosystem function.“

In the Conclusion, there is an error in the third sentence of the second paragraph. The correct sentence is:

“Zooplankton biomass production is best explained by a combination of functional and phylogenetic diversity, whereas suppression of phytoplankton production was best explained by phylogenetic diversity.”

## Supporting information

S6 Fig Structural equation model to predict zooplankton biomass. This model is not the most parsimonious but is shown because it includes all parameter types (zooplankton biomass, diversity, chlorophyll a, and environmental variables). Significant paths (*p < 0.05, **p < 0.01, ***p < 0.001) and their unstandardized parameter estimations are shown in black. Non-significant paths are shown in grey. Epsilons indicate error in endogenous variables. This diagram demonstrates that diversity was the most significant predictor of zooplankton biomass and was retained as significant when pathways from the environmental variables were included, as was the case in all models.
(TIF)

S7 Fig Structural equation model to predict chlorophyll a. This model is not the most parsimonious but is shown because it includes all parameter types (chlorophyll a, zooplankton biomass, diversity, and environmental variables). Significant paths (*p < 0.05, **p < 0.01, ***p < 0.001) and their unstandardized parameter estimations are shown in black. Epsilons indicate error in endogenous variables. Non-significant paths are shown in grey. This diagram demonstrates that diversity was the most significant predictor of chlorophyll a and was retained as significant when pathways from the environmental variables were included, as was the case in all models.
(TIF)

S1 Table Structural equation models for predicting zooplankton community biomass ranked in increasing order of AIC. Zooplankton community biomass (Z.bmass) and chlorophyll *a* (chl) were ln transformed. The environmental variables selected through multiple regression (Env) were elevation and log TP. PCA refers to the first two axes of a PCA of all standardized environmental variables.
(DOCX)

S2 Table Structural equation models for predicting chlorophyll *a* ranked in increasing order of AIC. Zooplankton community biomass (Z.bmass) and chlorophyll *a* (chl) and total phosphorous (TP) were ln transformed. The environmental variables selected through multiple regression (Env) were ln DIC, log area, ln depth, ln pH, ln wet days, and ln TP. PCA refers to the first two axes of a PCA of all standardized environmental variables.
(DOCX)

S1 File Supporting data. Zooplankton, chlorophyll *a*, environmental, spatial, trait, and phylogeny data for all ponds and species.
(XLS)
