# Supplementary figures and images for: Ecosystem Functions across Trophic Levels Are Linked to Functional and Phylogenetic Diversity
Source: PLoS One. 2015 Feb 18;10(2):e0117595. doi: 10.1371/journal.pone.0117595 (PMC4333825; doi:10.1371/journal.pone.0117595)

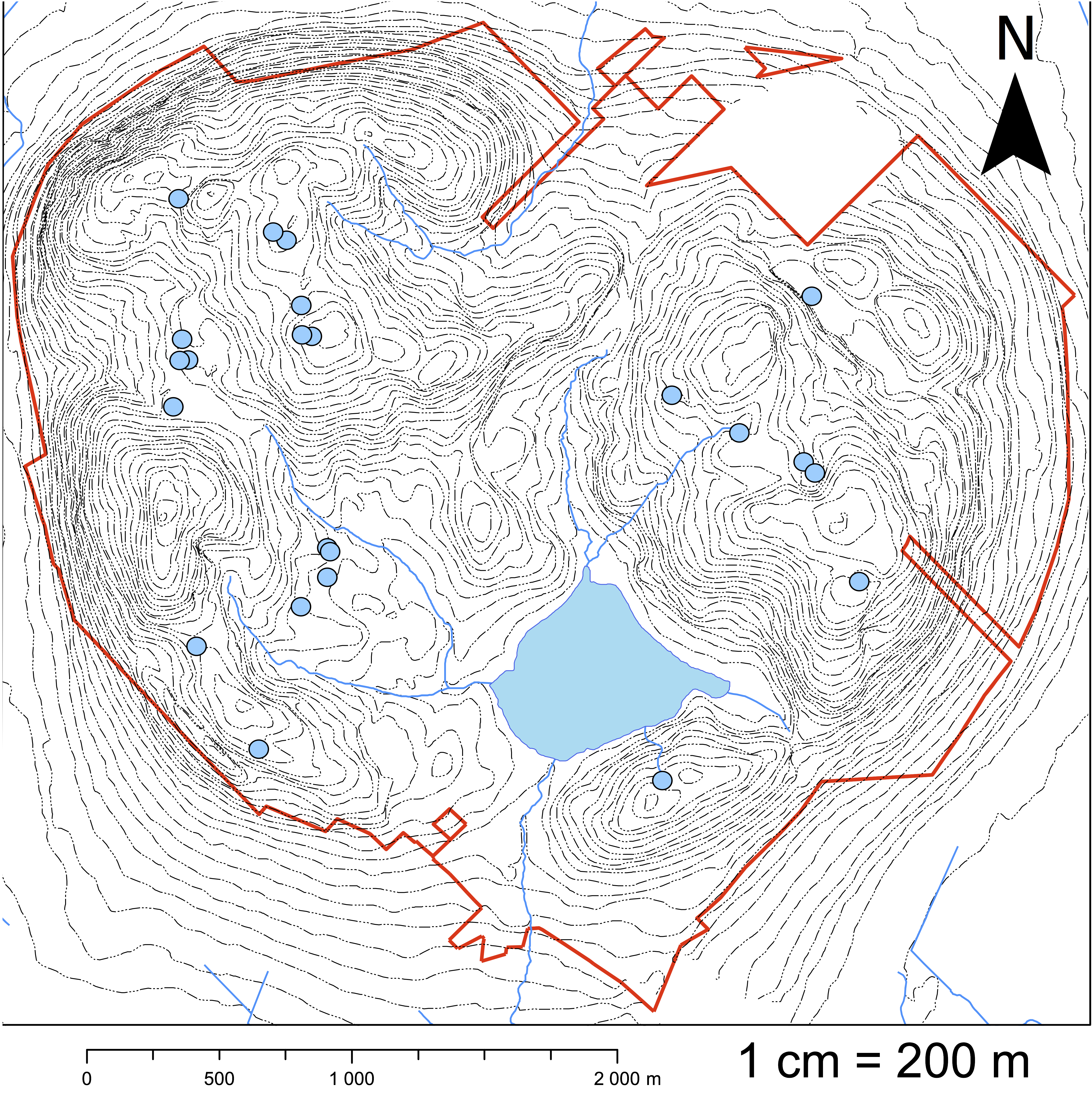

Supplement: S1 Fig — The red outline marks the Gault Nature Reserve border. Map courtesy of Gault Nature Reserve. (TIFF) [file pone.0117595.s001.tiff]

a)

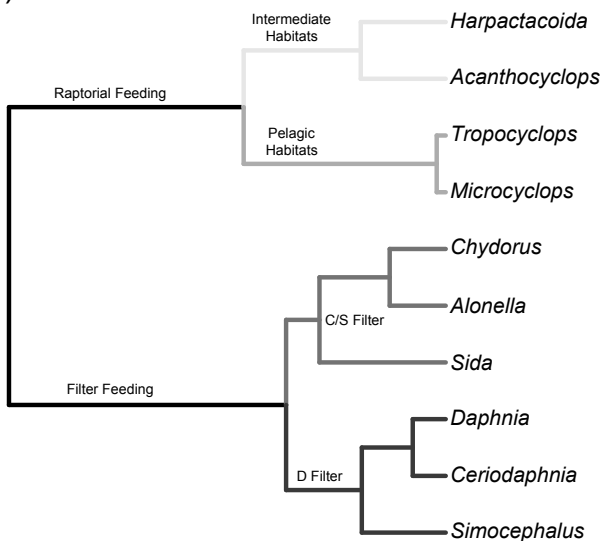

b)

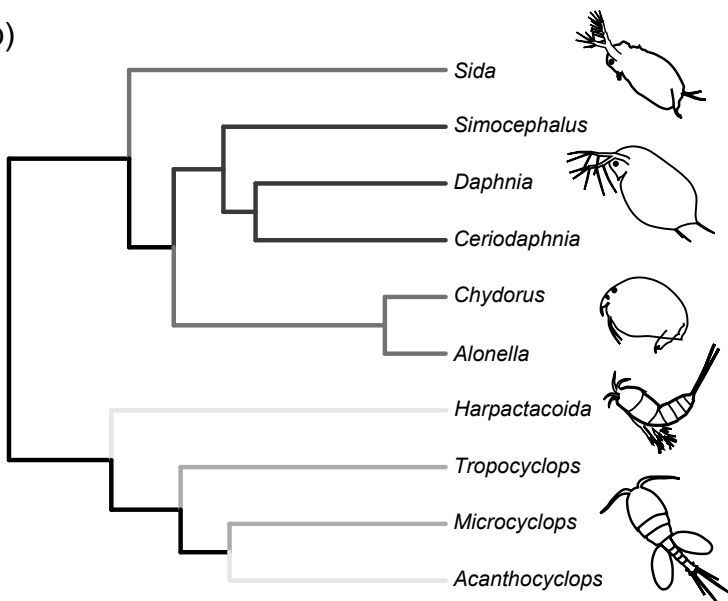

Supplement: S2 Fig — All five traits were used to create the functional dendrogram (a) and we have marked traits that divide clearly across the main functional bifurcations. The four functional groups selected from the trait dendrogram (a) are distinguished by shade and these are retained in the phylogenetic tree (b). (PDF) [file pone.0117595.s002.pdf]

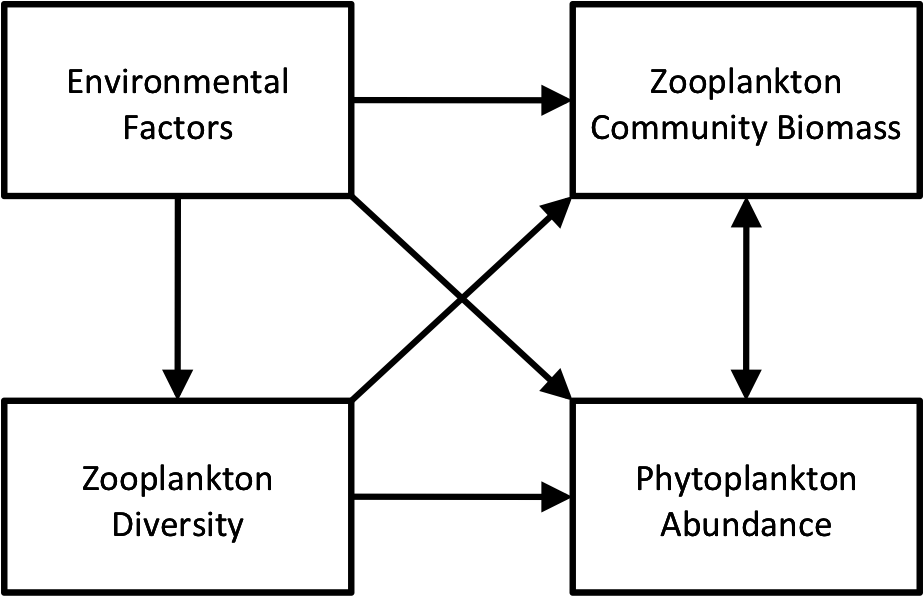

Supplement: S3 Fig — Note: because the link between phytoplankton abundance and zooplankton biomass is a trophic link, the direction of the arrow between these variables changes depending on which one we are trying to predict. (PNG) [file pone.0117595.s003.png]

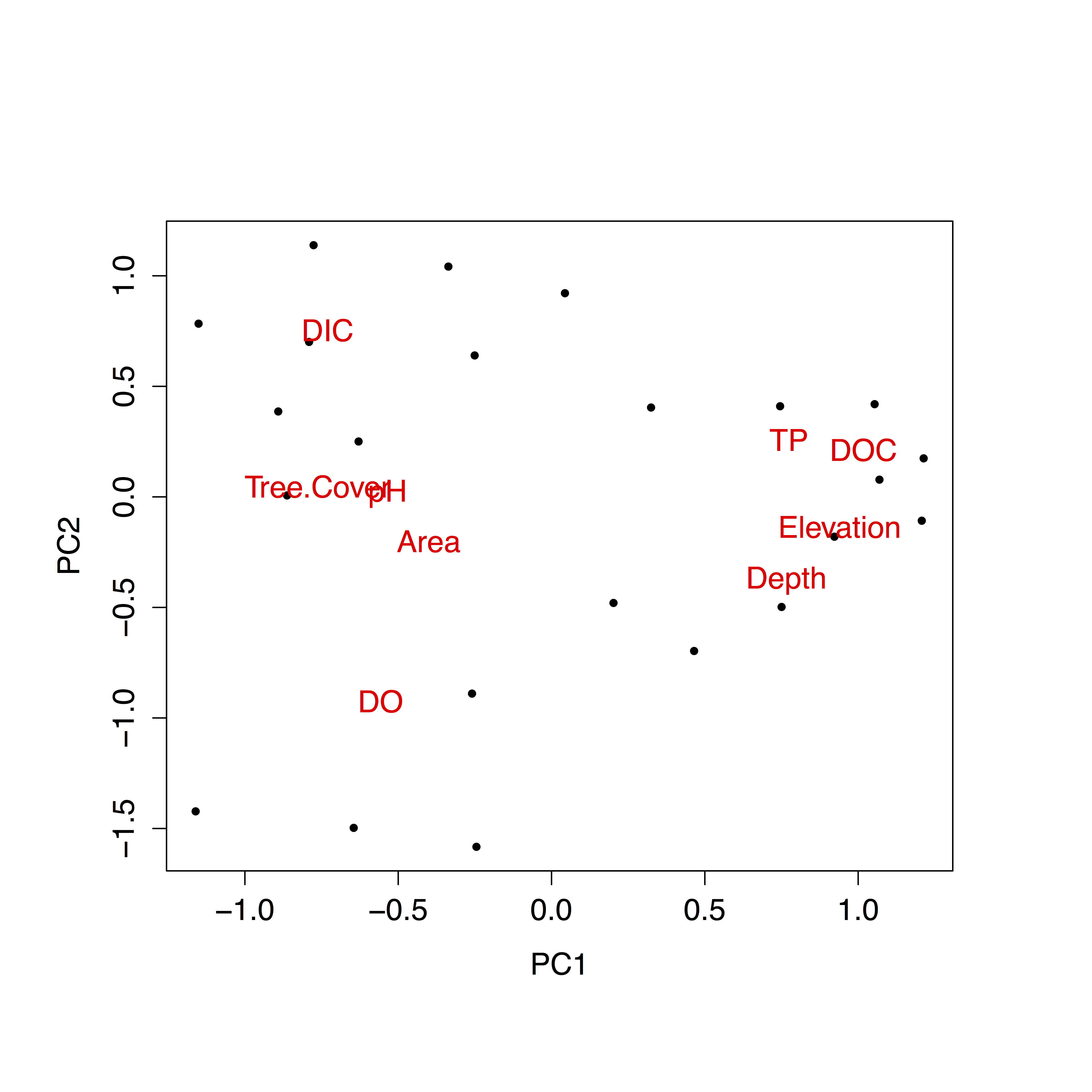

Supplement: S4 Fig — The black dots mark the position of the ponds in multivariate environmental space. All variables were standardized to a mean of zero and a standard deviation of one prior to calculating the PCA. (TIFF) [file pone.0117595.s004.tiff]

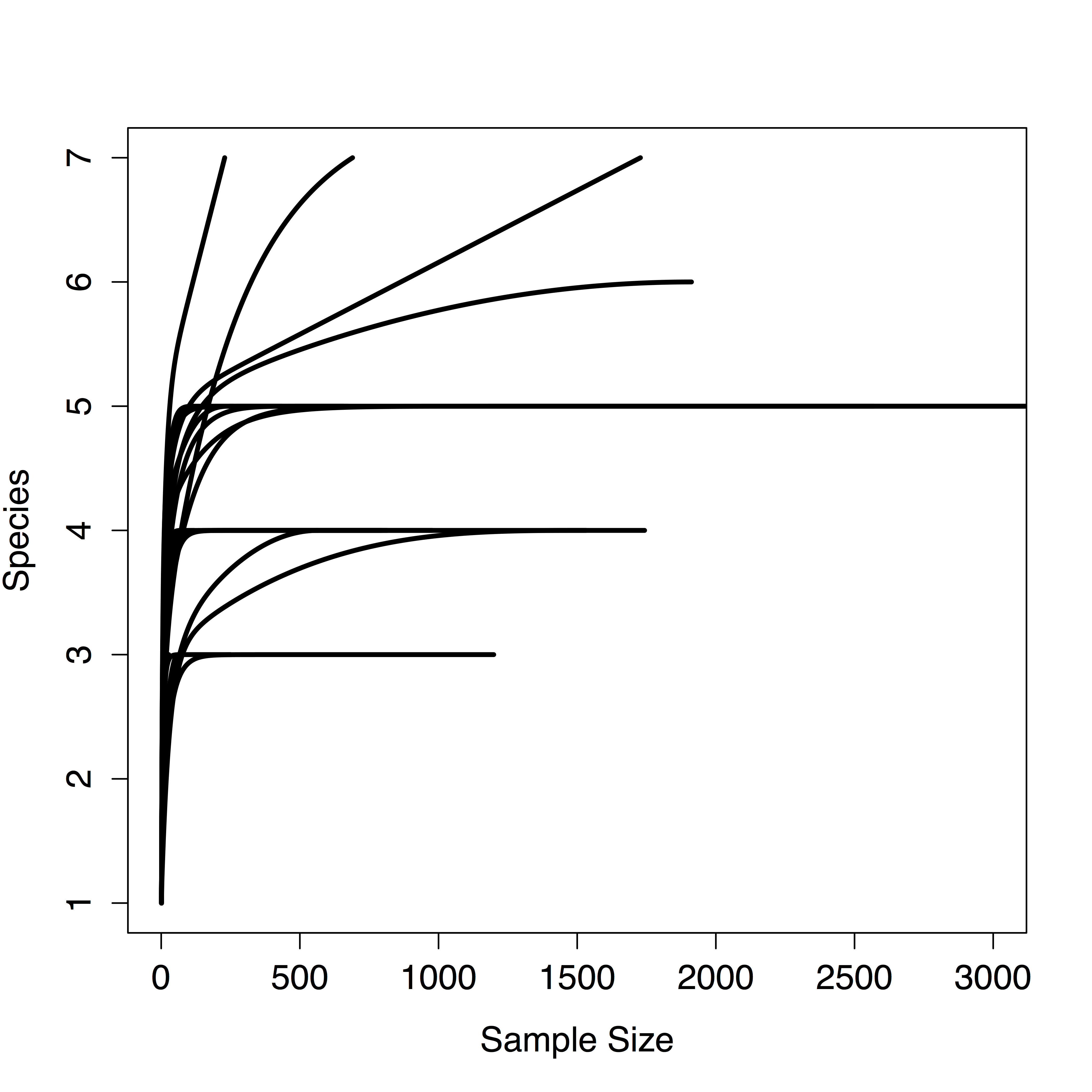

Supplement: S5 Fig — (TIFF) [file pone.0117595.s005.tiff]

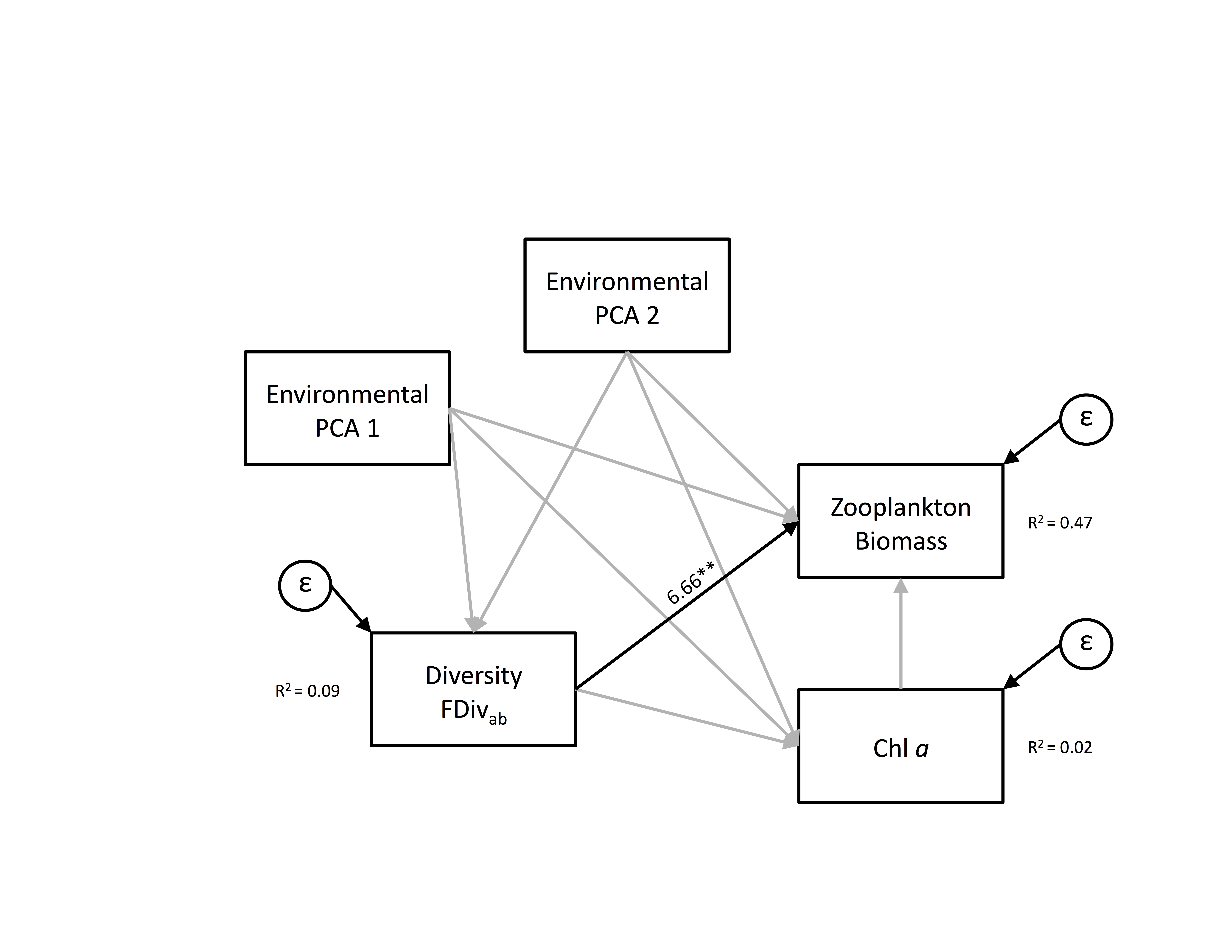

Supplement: S6 Fig — This model is not the most parsimonious, but is shown because it includes all parameter types (zooplankton biomass, diversity, chlorophyll a, and environmental variables). Significant paths (*p < 0.05, **p < 0.01, ***p < 0.001) and their unstandardized parameter estimations are shown in black. Non-significant paths are shown in grey. Epsilons indicate error in endogenous variables. This diagram demonstrates that diversity was the most significant predictor of zooplankton biomass and was retained as significant when pathways from the environmental variables were included, as was the case in all models. (TIFF) [file pone.0117595.s006.tiff]

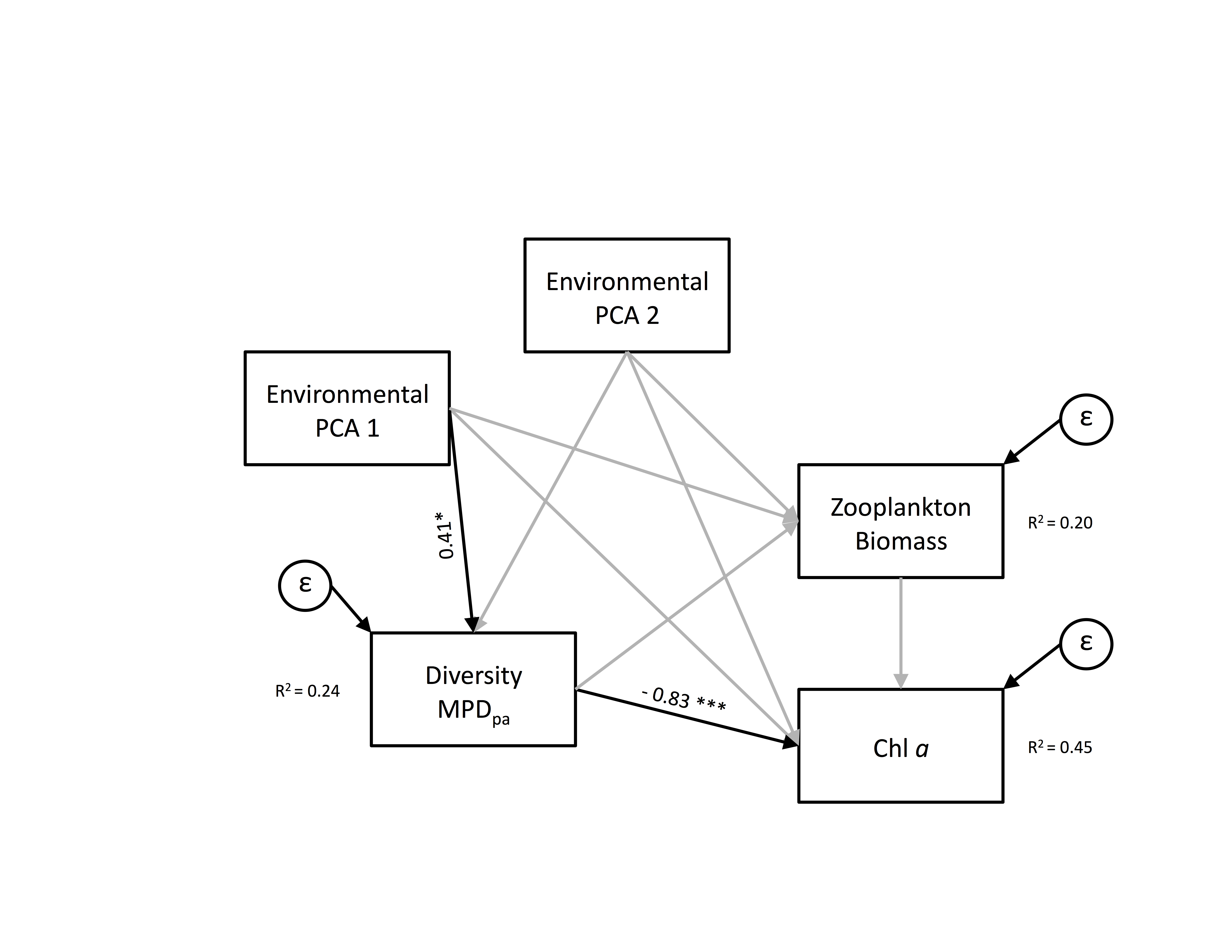

Supplement: S7 Fig — This model is not the most parsimonious, but is shown because it includes all parameter types (chlorophyll a, zooplankton biomass, diversity, and environmental variables). Significant paths (*p < 0.05, **p < 0.01, ***p < 0.001) and their unstandardized parameter estimations are shown in black. Epsilons indicate error in endogenous variables. Non-significant paths are shown in grey. This diagram demonstrates that diversity was the most significant predictor of chlorophyll a and was retained as significant when pathways from the environmental variables were included, as was the case in all models. (TIFF) [file pone.0117595.s007.tiff]
