# Supplementary material for: Ecosystem Functions across Trophic Levels Are Linked to Functional and Phylogenetic Diversity
Source: PLoS One. 2015 Feb 18;10(2):e0117595. doi: 10.1371/journal.pone.0117595 (PMC4333825; doi:10.1371/journal.pone.0117595)
Supplement: S1 Table — Zooplankton community biomass (Z.bmass) and chlorophyll a (chl) were ln transformed. The environmental variables selected through multiple regression (Env) were elevation, DIC, and log TP. PCA refers to the first two axes of a PCA of all standardized environmental variables. The χ2 test provides a test of how well the model fits the data. Models with p-values >0.05 are considered to be a reasonable fit to the data. Models are saturated when paths are specified between all variables and are considered to fit the data perfectly (Grace 2006). (DOCX) [file pone.0117595.s008.docx]

|  | Model | AIC | χ^2^ | d.f. | *P* value |
| --- | --- | --- | --- | --- | --- |
| 1 | Z.bmass ~ FDiv_ab_ | 37 | 0 | 0 | saturated |
| 2 | Z.bmass ~ FDiv_ab_ + chl | 103 | 0 | 0 | saturated |
| 3 | Z.bmass ~ chl | 146 | 0 | 0 | saturated |
| 4 | Z.bmass ~ FDiv_ab_ + PCA  FDiv_ab_ ~ PCA | 151 | 0 | 1 | 1.000 |
| 5 | Z.bmass ~ PCA | 189 | 0 | 0 | saturated |
| 6 | Z.bmass ~ FDiv_ab_ + PCA + chl  FDiv_ab_ ~ PCA  chl ~ FDiv_ab_ + PCA | 221 | 0 | 1 | 1.000 |
| 7 | Z.bmass ~ FDiv_ab_ + Env  FDiv_ab_ ~ Env | 234 | 4.03 | 2 | 0.134 |
| 8 | Z.bmass ~ PCA + chl  chl ~ PCA | 261 | 0 | 1 | 1.000 |
| 9 | Z.bmass ~ Env | 272 | 0 | 0 | saturated |
| 10 | Z.bmass ~ FDiv_ab_ + Env + chl  FDiv_ab_ ~ Env  chl ~ FDiv_ab_ + Env | 304 | 4.03 | 2 | 0.134 |
| 11 | Z.bmass ~ Env + chl  chl ~ Env | 342 | 4.02 | 2 | 0.134 |
