# Supplementary material for: Ecosystem Functions across Trophic Levels Are Linked to Functional and Phylogenetic Diversity
Source: PLoS One. 2015 Feb 18;10(2):e0117595. doi: 10.1371/journal.pone.0117595 (PMC4333825; doi:10.1371/journal.pone.0117595)
Supplement: S2 Table — Zooplankton community biomass (Z.bmass), chlorophyll a (chl), and total phosphorous (TP) were ln transformed. The environmental variables selected through multiple regression (Env) were % tree cover, DIC, log area, log depth, pH, and log DOC. PCA refers to the first two axes of a PCA of all standardized environmental variables. The χ2 test provides a test of how well the model fits the data. Models with p-values >0.05 are considered to be a reasonable fit to the data. Models are saturated when paths are specified between all variables and are considered to fit the data perfectly (Grace 2006). (DOCX) [file pone.0117595.s009.docx]

|  | Model | AIC | χ^2^ | d.f. | *P* value |
| --- | --- | --- | --- | --- | --- |
| 1 | chl ~ MPD_pa_ | 114 | 0 | 0 | saturated |
| 2 | chl ~ TP | 133 | 0 | 0 | saturated |
| 3 | chl ~ Z.bmass | 146 | 0 | 0 | saturated |
| 4 | chl ~ MPD_pa_ + TP  MPD_pa_ ~ TP | 173 | 0 | 0 | saturated |
| 5 | chl ~ PCA | 177 | 0 | 0 | saturated |
| 6 | chl ~ MPD_pa_ + Z.bmass  Z.bmass ~ MPD_pa_ | 197 | 0 | 0 | saturated |
| 7 | chl ~ MPD_pa_ + PCA  MPD_pa_ ~ PCA | 222 | 0 | 1 | 1.000 |
| 8 | chl ~ PCA + Z.bmass  Z.bmass ~ PCA | 256 | 0 | 1 | 1.000 |
| 9 | chl ~ MPD_pa_ + TP  MPD_pa_ ~ TP  Z.bmass ~ MPD_pa_ + TP | 258 | 0 | 0 | saturated |
| 10 | chl ~ MPD_pa_ + PCA + Z.bmass  MPD_pa_ ~ PCA  Z.bmass ~ MPD_pa_ + PCA | 305 | 0 | 1 | 1.000 |
| 11 | chl ~ Env | 515 | 0 | 0 | saturated |
| 12 | chl ~ MPD_pa_ + Env  MPD_pa_ ~ Env | 567 | 14.41 | 6 | 0.017 |
| 13 | chl ~ Env + Z.bmass  Z.bmass ~ Env | 600 | 15.41 | 6 | 0.017 |
| 14 | chl ~ MPD_pa_ + Env +Z.bmass  MPD_pa_ ~ Env  Z.bmass ~ MPD_pa_ + Env | 648 | 15.41 | 6 | 0.017 |
