# Supplementary figures and images for: Correction: Ecosystem Functions across Trophic Levels Are Linked to Functional and Phylogenetic Diversity
Source: PLoS One. 2019 Jul 18;14(7):e0220213. doi: 10.1371/journal.pone.0220213 (PMC6638964; doi:10.1371/journal.pone.0220213)

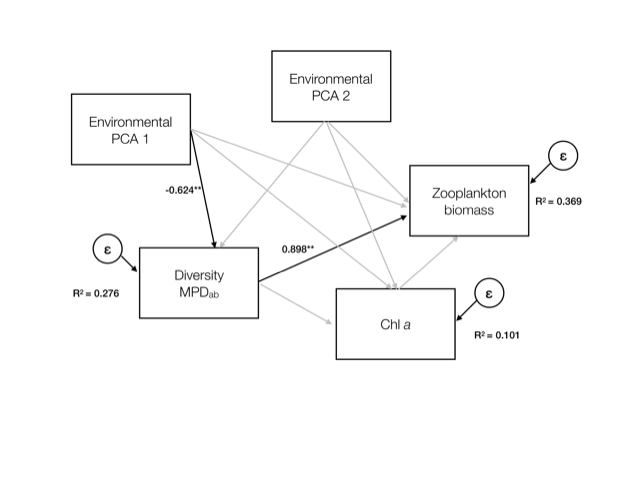

Supplement: S6 Fig — This model is not the most parsimonious but is shown because it includes all parameter types (zooplankton biomass, diversity, chlorophyll a, and environmental variables). Significant paths (*p < 0.05, **p < 0.01, ***p < 0.001) and their unstandardized parameter estimations are shown in black. Non-significant paths are shown in grey. Epsilons indicate error in endogenous variables. This diagram demonstrates that diversity was the most significant predictor of zooplankton biomass and was retained as significant when pathways from the environmental variables were included, as was the case in all models. (TIF) [file pone.0220213.s001.tif]

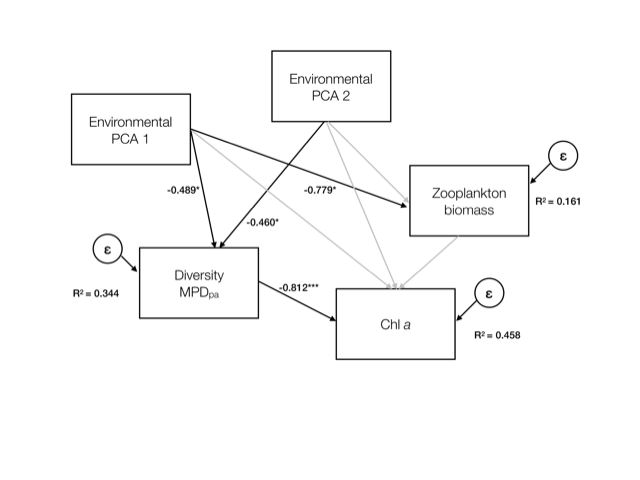

Supplement: S7 Fig — This model is not the most parsimonious but is shown because it includes all parameter types (chlorophyll a, zooplankton biomass, diversity, and environmental variables). Significant paths (*p < 0.05, **p < 0.01, ***p < 0.001) and their unstandardized parameter estimations are shown in black. Epsilons indicate error in endogenous variables. Non-significant paths are shown in grey. This diagram demonstrates that diversity was the most significant predictor of chlorophyll a and was retained as significant when pathways from the environmental variables were included, as was the case in all models. (TIF) [file pone.0220213.s002.tif]
