# Supplementary material for: Correction: Ecosystem Functions across Trophic Levels Are Linked to Functional and Phylogenetic Diversity
Source: PLoS One. 2019 Jul 18;14(7):e0220213. doi: 10.1371/journal.pone.0220213 (PMC6638964; doi:10.1371/journal.pone.0220213)
Supplement: S1 Table — Zooplankton community biomass (Z.bmass) and chlorophyll a (chl) were ln transformed. The environmental variables selected through multiple regression (Env) were elevation and log TP. PCA refers to the first two axes of a PCA of all standardized environmental variables. (DOCX) [file pone.0220213.s003.docx]

|  | Model | AIC |
| --- | --- | --- |
| 1 | Z.bmass ~ FDiv_ab_ | 146 |
| 3 | Z.bmass ~ chl | 155 |
| 5 | Z.bmass ~ PCA | 205 |
| 9 | Z.bmass ~ Env | 213 |
| 2 | Z.bmass ~ FDiv_ab_ + chl | 213 |
| 4 | Z.bmass ~ FDiv_ab_ + PCA  FDiv_ab_ ~ PCA | 262 |
| 7 | Z.bmass ~ FDiv_ab_ + Env  FDiv_ab_ ~ Env | 270 |
| 8 | Z.bmass ~ PCA + chl  chl ~ PCA | 274 |
| 11 | Z.bmass ~ Env + chl  chl ~ Env | 283 |
| 6 | Z.bmass ~ FDiv_ab_ + PCA + chl  FDiv_ab_ ~ PCA  chl ~ FDiv_ab_ + PCA | 333 |
| 10 | Z.bmass ~ FDiv_ab_ + Env + chl  FDiv_ab_ ~ Env  chl ~ FDiv_ab_ + Env | 341 |
|  |  |  |
