# Supplementary material for: Correction: Ecosystem Functions across Trophic Levels Are Linked to Functional and Phylogenetic Diversity
Source: PLoS One. 2019 Jul 18;14(7):e0220213. doi: 10.1371/journal.pone.0220213 (PMC6638964; doi:10.1371/journal.pone.0220213)
Supplement: S2 Table — Zooplankton community biomass (Z.bmass) and chlorophyll a (chl) and total phosphorous (TP) were ln transformed. The environmental variables selected through multiple regression (Env) were ln DIC, log area, ln depth, ln pH, ln wet days, and ln TP. PCA refers to the first two axes of a PCA of all standardized environmental variables. (DOCX) [file pone.0220213.s004.docx]

|  | Model | AIC |
| --- | --- | --- |
| 1 | chl ~ MPD_pa_ | 118 |
| 2 | chl ~ TP | 133 |
| 3 | chl ~ Z.bmass | 155 |
| 5 | chl ~ PCA | 184 |
| 4 | chl ~ MPD_pa_ + TP  MPD_pa_ ~ TP | 188 |
| 6 | chl ~ MPD_pa_ + Z.bmass  Z.bmass ~ MPD_pa_ | 209 |
| 7 | chl ~ MPD_pa_ + PCA  MPD_pa_ ~ PCA | 231 |
| 9 | chl ~ MPD_pa_ + TP + Z.bmass  Z.bmass ~ MPD_pa_ + TP  MPD_pa_ ~ TP | 274 |
| 8 | chl ~ PCA + Z.bmass  Z.bmass ~ PCA | 274 |
| 10 | chl ~ MPD_pa_ + PCA + Z.bmass  MPD_pa_ ~ PCA  Z.bmass ~ MPD_pa_ + PCA | 321 |
| 11 | chl ~ Env | 389 |
| 12 | chl ~ MPD_pa_ + Env  MPD_pa_ ~ Env | 442 |
| 13 | chl ~ Env + Z.bmass  Z.bmass ~ Env | 477 |
| 14 | chl ~ MPD_pa_ + Env +Z.bmass  MPD_pa_ ~ Env  Z.bmass ~ MPD_pa_ + Env | 531 |
